# Type 2 diabetes progression differently affects endothelial function and vascular contractility in the aorta and the pulmonary artery

**DOI:** 10.1038/s41598-021-85606-7

**Published:** 2021-03-15

**Authors:** Bernardete F. Melo, Jesus Prieto-Lloret, Marlene D. Cabral, Fatima O. Martins, Inês B. Martins, Joana F. Sacramento, Pedro Ruivo, Tânia Carvalho, Silvia V. Conde

**Affiliations:** 1 iNOVA4Health, CEDOC, NOVA Medical School, NMS, Faculdade Ciências Médicas, Universidade Nova de Lisboa, Rua Camara Pestana, nº6, 6A, edificio II, piso 3, 1150-082 Lisbon, Portugal; 2 Instituto de Medicina Molecular João Lobo Antunes, Lisbon, Portugal; 3 Champalimaud Research and Clinical Centre, Champalimaud Centre for the Unknown, Lisbon, Portugal

**Keywords:** Diseases, Endocrinology, Medical research, Pathogenesis, Physiology, Circulation, Metabolism

## Abstract

Type 2 diabetes (T2D) is associated with cardiovascular and pulmonary disease. How T2D affects pulmonary endothelial function is not well characterized. We investigated the effects of T2D progression on contractility machinery and endothelial function in the pulmonary and systemic circulation and the mechanisms promoting the dysfunction, using pulmonary artery (PA) and aorta. A high-fat (HF, 3 weeks 60% lipid-rich diet) and a high-fat/high-sucrose (HFHSu, combined 60% lipid-rich diet and 35% sucrose during 25 weeks) groups were used as prediabetes and T2D rat models. We found that T2D progression differently affects endothelial function and vascular contractility in the aorta and PA, with the contractile machinery being altered in the PA and aorta in prediabetes and T2D animals; and endothelial function being affected in both models in the aorta but only affected in the PA of T2D animals, meaning that PA is more resistant than aorta to endothelial dysfunction. Additionally, PA and systemic endothelial dysfunction in diabetic rats were associated with alterations in the nitrergic system and inflammatory pathways. PA dysfunction in T2D involves endothelial wall mineralization. The understanding of the mechanisms behind PA dysfunction in T2D can lead to significant advances in both preventative and therapeutic treatments of pulmonary disease-associated diabetes.

## Introduction

The endothelium is a physical barrier between blood and tissues, therefore controlling the exchange of molecules between them^1^. In addition, endothelial cells produce and release several mediators, including vasodilators, as nitric oxide (NO) and prostacyclin, vasoconstrictors, as endothelin-1 and prostaglandin F_2α_ (PGF_2α_), and substances that participate in coagulation, fibrinolysis and inflammatory and immunological reactions as well as reactive oxygen species (ROS) and growth factors promoting cell growth, among others. Any disturbances affecting the endothelium as, for example, alterations in the capacity to produce and release these substances, or alterations in the function of the endothelium as physical barrier may cause endothelial dysfunction^2^.

Endothelial dysfunction is characterized by a reduction of the bioavailability of vasodilators whereas endothelium-derived contracting factors are increased^3^. This imbalance leads to vasoconstriction, leucocyte adherence, vascular inflammation, atherosclerosis and thrombosis, among others^4^.

Impaired endothelium-dependent relaxation is well documented in type 2 diabetes (T2D), which is mainly caused by reduced NO bioavailability. Other factors that can contribute to endothelial dysfunction in T2D include a decrease in the production of other relaxing factors, an increase in the production of vasoconstrictor substances or a reduction in the sensitivity of vascular smooth muscle (VSM) to relaxing factors^5^. One of the pathological characteristics of T2D is hyperglycemia together with insulin resistance and, it is known that vascular endothelial cells are very prone to be damaged by hyperglycemic stress^6^. One of the mechanisms contributing to hyperglycemic-vascular damage is the increased production of advanced glycation end products (AGEs) that by biding to their cellular receptors augment oxidant stress and induce a state of endothelial cell activation contributing to endothelial impairment^7,8^. Hyperglycemia also contributes to alterations in the balance between vasodilators and vasoconstrictors, as it enhances the secretion, in vitro, of endothelin-1 and decreases NO production in the aorta of diabetic rats and coronary microvessels in humans^9^. IL-1 and IL-6, proinflammatory signaling molecules, also showed to play an important role in mediating vascular endothelial dysfunction, by exacerbating oxidative stress and reducing phosphorylation of eNOS^10^.

Diabetes is a major risk factor of peripheral artery disease, leading to an accelerated disease course and several complications^11^. More recently, a link between diabetes, especially T2D, and the development of pulmonary vascular disease, particularly pulmonary arterial hypertension (PAH) which can lead to right ventricular failure and death^12–14^ was suggested. Furthermore, it has been recognized that these disorders, as well as systemic hypertension, are associated with impaired vasorelaxation^12–15^, diabetes mellitus, obesity, hypercholesterolemia or a sedentary lifestyle, enhancing the risk for endothelial dysfunction, atherosclerosis and systemic cardiovascular diseases^16^.

The role of prediabetes and diabetes in the development and progression of vascular dysfunction and its complications is well documented in several arterial types and organs^17^. However, there is less information available regarding the effects of these disorders on pulmonary vascular function^18^. Taking this into account, we have investigated the effect of different stages of T2D on the contractile machinery and endothelial function in the pulmonary and systemic circulation and the molecular players involved. We observed that T2D progression differently affects endothelial function and vascular contractility in the aorta and PA, with diabetes affecting endothelial function earlier in the aorta than in the PA, suggesting that the latter is more resistant to diabetes-induced endothelial dysfunction. In the aorta we confirmed the well documented relationship between the nitrergic system—inflammation—oxidative stress and endothelial dysfunction while in the PA despite the involvement of all these mechanisms, a delayed disruption of the nitrergic system could protect these vessels in initial stages of dysmetabolism. Additionally, we found that in later stages of metabolic disease, in T2D animals, wall mineralization might contribute to aggravated endothelial dysfunction in the PA.

## Methods

### Ethical approval

All experiments and animal care were performed in accordance with the European Union Directive for Protection of Vertebrates Used for Experimental and Other Scientific Ends (2010/63/EU) and with the ARRIVE guidelines. Experimental protocols were approved by the Ethics Committee of NOVA Medical School/Faculdade de Ciências Médicas (nº18/2016/CEFCM) and by the Portuguese Authority for the animal Health (DGAV, Ref 0421/000/000/2016).

### Animals and surgical procedures

Experiments were performed in 8 weeks old male Wistar rats (200–490 g), obtained from the vivarium of NOVA Medical School, Faculdade de Ciências Médicas. Animals were housed in a controlled environment (21 ± 1 °C; 55 ± 10% humidity) with a 12-h light/dark cycle and free access to food and water. In this project we have used two diet-induced animal models: a prediabetes model obtained by submitting animals to a high-fat (HF) diet and an early-phase type 2 diabetes model obtained by submitting animals to a high-fat and high-sucrose (HFHSu) diet. These animal models were compared with a control group that fed a standard diet (7.4% fat plus 75% carbohydrate [4% sugar] plus 17% protein; SDS diets RM1; Probiológica, Lisbon, Portugal; For a more detailed description see Table S1). The HF model fed a lipid-rich diet with 60% of energy from fat (61.6% fat + 20.3% carbohydrate + 19.1% protein; Mucedola, Milan, Italy; For a more detailed description see Table S2), during 3 weeks and the HFHSu model was obtained by a combination of a lipid-rich diet (61.6% fat + 20.3% carbohydrate + 19.1% protein; Mucedola, Milan, Italy) plus 35% (wt/vol.) sucrose (PanReac, Madrid, Spain), during 25 weeks. Body weight was weekly recorded meanwhile caloric and liquid intake were monitored daily in all groups of animals. Metabolic parameters as fasting glycemia, insulin sensitivity and glucose tolerance were monitored throughout the experimental period by an insulin tolerance test (ITT) and an oral glucose tolerance test (OGTT), respectively. At the end of the experimental period, the rats were anesthetized with sodium pentobarbital (60 mg/kg, i.p.) and plasma was collected by heart puncture and then processed to measure NO levels. The aorta and the pulmonary arteries were excised and dissected for vascular function studies or stored for posterior analyses.

### Insulin tolerance test

Insulin sensitivity was assessed by an ITT in conscious animals after an overnight fast. Briefly, the ITT was performed by administrating a bolus of insulin (0.1U/Kg) in the tail vein^19^ and measuring of the decline of glycemia in the next 15 min. Blood was collected via tail tipping and glycemia evaluated with a glucometer (Precision Xtra Meter, Abbott Diabetes Care, Portugal) and test strips (Abbott Diabetes Care, Portugal). The constant rate for plasma glucose decline (K_ITT_) was calculated as previously described^19^.

### Oral glucose tolerance test

OGTT evaluated the insulin released and the sensitivity of the peripheral tissue toward the insulin action. For the OGTT, the animals were fasted overnight and in the morning a bolus of glucose (2 g/kg) was administrated intragastrically by oral gavage^20^. Blood samples were collected by tail tipping before (0 min) and 15, 30, 60, 120, 180 min after glucose administration. Glucose levels were measured as for the ITT. The product of the area under the curve (AUC) was used to estimate the glucose tolerance.

### Plasma insulin levels measurement

Plasma insulin levels were determined with an enzyme-linked immunosorbent assay (ELISA) kit Mercodia Ultrasensitive Rat Insulin ELISA Kit (Mercodia AB, Uppsala, Sweden) as previously described^19^.

### Artery mounting and measurement of tension development

The physiological function of the arteries was studied using a small vessel wire myograph (DMT, Denmark), technique that allows ex vivo monitoring of isometric tension in response to different pathophysiological stimuli^21^. The bath chambers for isolated arteries were maintained in normoxic conditions (gassing with a mixture of 5%CO_2_/21%O_2_/rest N_2_) and at 37 °C throughout the whole experimental procedure. Fresh conduit (aortic) and resistance (pulmonary) blood vessels were dissected in cold physiological salt solution (PSS) that contained the following (in mM): 118 NaCl, 24 NaHCO_3_, 1 MgSO_4_, 0.435 NaH_2_PO_4_, 5.56 glucose, 1.8 CaCl_2_ and 4 KCl. Rings of aorta or PA were dissected under a dissection microscope and withdrawn of all adventitia and parenchyma. The arteries were mounted to measure changes in isometric tension using a force transducer, stabilized for 30 min and then stretched to give a basal tension of 5–6mN. To check the viability of the vessels were used three responses (3 min each) of PSS containing 80 mmol/L KCl (KPSS, isotonic replacement of NaCl by KCl), washing twice with PSS between them.

After washing out, arteries were constricted with cumulative concentrations of PGF_2α_ (0.03–10 µM or 30 µM; Bio-Techne, MN, USA) to study their contractile properties. In a subsequent step, selecting a sub-maximal PGF_2α_ concentration (according with the previous dose response curve), the vasorelaxant effect of acetylcholine was recorded (0.03–30 µM; Sigma-Aldrich, Madrid, Spain), as an index of endothelial integrity. Additionally, and to further study the contractile properties of the arteries a 1 µM concentration of phenylephrine (PE, Sigma-Aldrich, Madrid, Spain) was added.

### Western blot analyses of eNOS, iNOS, PGF_2α_ receptors, AGEs receptors, IL-1 and IL-6 receptors and catalase

For western blot analysis, approximately 10 mg of PA or aorta arteries were dissected and homogenized in liquid nitrogen and then placed in approximately 350ul of Zurich medium containing proteases inhibitors. Proteins (20 μg) were separated by electrophoresis in 10% sodium dodecylsulfate-polyacrylamide gel electrophoresis (SDS-PAGE), followed by transference to a nitrocellulose membrane (BioRad, Germany). The membranes were then blocked in I-Block solution (Applied Biosystems, Foster City, USA) during 1 h. After blocking, membranes were incubated overnight, at 4 °C, with the following primary antibodies: monoclonal mouse anti-eNOS (1:500; Santa Cruz Biotechnology, Madrid, Spain), polyclonal rabbit anti-iNOS (1:100; Santa Cruz Biotechnology, Madrid, Spain), polyclonal rabbit anti-Prostaglandin F2 alpha Receptor antibody (1:200; Santa Cruz Biotechnology, Madrid, Spain); polyclonal rabbit anti-AGEs receptor (1:500; TransGenic Inc., Kobe, Japan), polyclonal mouse anti-IL-1 receptor (1:50; Santa Cruz Biotechnology, Madrid, Spain), polyclonal mouse anti-IL-6 receptor (1:50; Santa Cruz Biotechnology, Madrid, Spain) or polyclonal goat anti-Catalase antibody (1:500; SICGEN, Coimbra, Portugal). Afterwards, membranes were incubated with biotin-conjugated goat anti-rabbit IgG (1:10,000; Millipore, Madrid, Spain), mouse anti-goat IgG (1:10,000; Millipore, Madrid, Spain) or goat anti-mouse IgG (1:10,000; Millipore, Madrid, Spain), at room temperature for 90 min, and with horseradish peroxidase-conjugated streptavidin (1:10,000, Thermo Scientific, USA), at room temperature for 30 min. Between incubations membranes were washed with TBS-T (0,01%) for 3 times, 5 min. Chemiluminescence signals were developed with enhanced chemiluminescence reagent (Clarity Western ECL, Bio-Rad, United States), the signal detected in a ChemiDoc Touch Imaging System (Chemidoc; BioRad, Madrid, Spain) and quantified using the Image Lab software (BioRad). The membranes were reprobed with goat anti-calnexin (1:1000; SicGen, Coimbra, Portugal), goat anti-β-Actin (1:1000; Santa Cruz Biotechnology, Madrid, Spain) or goat anti-GAPDH (1:1000; Santa Cruz Biotechnology, Madrid, Spain) to compare and normalize the expression of proteins with the amount of protein loaded.

### AGEs (anti N-(carboxyethyl) lysine quantification

Pulmonary and aorta arteries samples lysates were prepared as previously described for western blot analysis. For dot-blot method 5ug of protein from each sample was applied directly on nitrocellulose membrane on the dot-blot vacuum system and each well was washed with TBST (137 mM NaCl, 2.7 mM KCl, 20 mM Tris, pH7.4, plus 0.1% Tween-20). The membrane was then removed from the dot-blot system and incubated with blocking buffer (BSA 5% in TBST) for 1 h at room temperature in a shaker. The membrane was incubated with primary antibody for AGEs (anti N-(carboxyethyl) lysine monoclonal antibody (1:500; Cosmobio Co., Ltd, Japan) overnight at 4 °C. The membrane was washed with TBS-T (0.01%) for 3 times, 5 min, and incubated with goat anti-mouse-HRP conjugated antibody (1:5000; Santa Cruz Biotechnology, Madrid, Spain) for 1 h at room temperature. Membrane was washed again and incubated with ECL substrate solution (Clarity Western ECL, Bio-Rad, United States) for chemiluminescence signal quantification in a ChemiDoc Touch Imaging System (Chemidoc; BioRad, Madrid, Spain). For detection and quantification of the loading control the membrane was stripped off, washed TBS-T (0,01%) for 4 times and loading control was evaluated by incubating the membrane with primary antibody goat anti-calnexin (1:1000; SicGen, Coimbra, Portugal) for 1 h at room temperature, washed 3 times with TBS-T (0,01%) and incubated with mouse anti-goat (1:5000; BioRad, Madrid, Spain). Chemiluminescence signals were developed with enhanced chemiluminescence reagent (Clarity Western ECL, Bio-Rad, United States) and the signal detected in a ChemiDoc Touch Imaging System (Chemidoc; BioRad, Madrid, Spain) for chemiluminescence signal quantification using the Image Lab software (BioRad).

### Nitric oxide and PGF_2α_ quantification

To quantify NO in pulmonary artery and in the aorta, the arteries were dissected, homogenized in liquid nitrogen and subsequently transferred to a homogenization buffer (25 mM Tris HCL, 1 mM EDTA, 1 mM EGTA). Afterwards the homogenates were centrifuged at 13000 g (4 °C) for 20 min and the supernatant collected. For plasma NO quantification, blood was collected from a heart puncture to EDTA-precoated tubes and centrifuged at 3000 g (4 °C) during 10 min. Plasma and artery homogenates were then deproteinized by diluting the samples with ethanol absolute at 4 °C (1:3). After 30 min on ice, samples were centrifuged in a microfuge (Eppendorf, Madrid, Spain) at 12000 g for 15 min. NO/NO^3−^ concentration was determined by using a specific and sensitive NO/ozone chemiluminescence technique (NO-Analyzer 280, Sievers Research Inc., Boulder Colorado). For plasma PGF_2α_ quantification, blood samples were treated as mentioned above. Since some samples normally have very low levels of PGF_2α_ extraction with a C18 reverse phase column was performed for a more accurate measurement. After processing the samples, PGF_2α_ levels were measured through an in vitro competitive ELISA (Enzyme-Linked Immunosorbent Assay) kit (Abcam, Cambridge, United Kingdom).

### Histochemical analysis of vascular mineralization

Aortas and PA were collected, dissected and immersion-fixed in PFA 4%. Samples were then embedded into paraffin (Sakura Finetek Europe B.V., Zoeterwoude, Netherlands) and longitudinal serial sections of 3 μm thick were obtained with a Microtome Microm HM200 (MICROM Laborgeräte GmbH, ZEISS Group, Walldorf, Germany). After sectioned, the samples were transferred into slides and stained with hematoxylin and eosin, Masson's trichrome (for collagen evaluation) and Taenzer-Unna Orcein (for elastin evaluation).

Aorta and PA lesions were examined by two pathologists blinded to experimental groups to avoid evaluation biases and classified according to previously published criteria^22,23^. A semiquantitative score of Hematoxylin–Eosin staining slides was determined based on calcification in arterial cross section according to a 4-tier severity scale: 0, no calcification; 1, focal calcification spots; 2, partial calcification covering 20–80% of the arterial circumference; and 3, circumferential calcification^24^. Cases with discrepancies were jointly re-evaluated until a consensus was reached. Representative photographs were acquired using NDP.view2 software (Hamamatsu, Japan) in slides digitally scanned in the Hamamatsu NanoZoomerSQ (Hamamatsu, Japan).

### Statistical analysis

All data obtained for the present work was evaluated using Graph Pad Prism Software, version 8 (GraphPad Software Inc., USA) and presented as scatter plots or mean values with standard deviation (SD). Significance of the differences between the mean values was calculated by one-and two-way ANOVA with Bonferroni multiple comparison test. Differences were considered significant at *p* < 0.05.

## Results

### Effect of hypercaloric diets on metabolic variables

We have used two different types of hypercaloric diets, a 3 weeks HF diet and 25 weeks of HFHSu to achieve different stages of metabolic dysfunction in rats, namely a prediabetes stage and an early stage of type 2 diabetes, respectively. Metabolic parameters and evaluation are summarised in Table 1. HF and HFHSu diet promoted a 24.7% and 102.6% increase in the caloric intake per day (control animals = 147.8 ± 816.5 kcal/day/kg), respectively, being this augmented intake reflected in animals weight gain: HF animals increased 0.26 ± 0.04 g/day and HFHSu animals 0.35 ± 0.03 g/day during diet period (weight gain control animals = 0.13 ± 0.04 g/day). As expected, although HF diet induced a state of metabolic dysfunction, the metabolic alterations promoted by this diet are less intense than the alterations promoted by HFHSu diet: HF diet was incapable of promoting an increase in fasting glycemia, while HFHSu increased significantly glycemia values by 17.3%; HF diet decreased insulin sensitivity by 61.6% and increased glucose intolerance by 20.8% and plasma insulin levels by 67.7% while HFHSu diet exert more pronounced effects on insulin sensitivity, plasma insulin levels and glucose tolerance in comparison with the controls (Table 1).

**Table 1:** Effect of hypercaloric diets, high-fat (HF) and high fat-high sucrose (HFHSu), on caloric intake, weight gain, insulin sensitivity and glucose tolerance in rats.

	CTL	HF	HFHSu
Weight increase (g/day)	0.13 ± 0.04	0.26 ± 0.04***	0.35 ± 0.03****^; ##^
Caloric Intake (Kcal/day/kg)	147.8 ± 16.5	184.3 ± 6.89**	299.4 ± 18.08****^; ####^
Fasting Glycemia (mg/dl)	86.4 ± 5.08	85.2 ± 3.56	101.3 ± 7.87**
Glucose tolerance AUC glycemia (mg/dl*min)	20,063 ± 1271	24,234 ± 1183****	25,392 ± 514.2****
Insulin sensitivity KITT (% glucose/min)	4.09 ± 0.42	1.57 ± 0.59****	1.31 ± 0.12****
Insulin levels (pmol/l)	122.1 ± 75.25	378.4 ± 181.50*	737.1 ± 193.50***^,#^

Animals were submitted to HF diet (60% lipid-rich diet) during 3 weeks and to HFHSu (60% lipid-rich diet plus 35% sucrose in drinking water) during 25 weeks. Insulin sensitivity was expressed as the constant of the insulin tolerance test, K_ITT_. Glucose tolerance was evaluated by an OGTT and depicted as the area under the curve of the glucose excursion curve. Results are presented as mean ± SD. One-way ANOVA with Dunnet’s Multiple Comparison test: *p < 0.05, **p < 0.01, ***p < 0.001 and ****p < 0.0001 comparing hypercaloric diet groups with control group; ^#^*p* < 0.05, ^##^*p* < 0.01 and ^####^*p* < 0.0001 comparing HF and HFHSu groups.

### Effect of hypercaloric diets on vascular contractility and endothelial function

**Figure 1:**
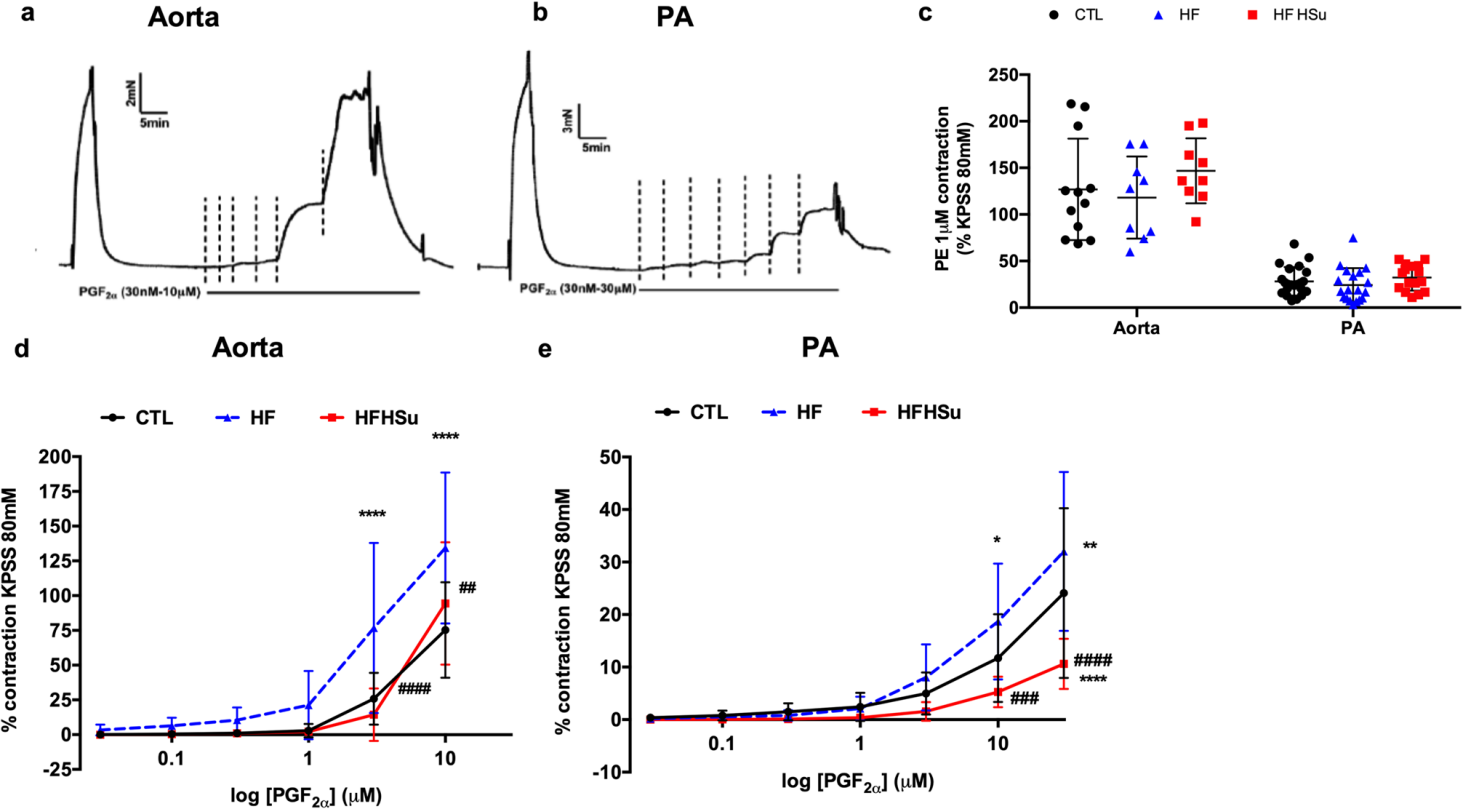
Effect of hypercaloric diets on vascular contractility in aorta and pulmonary artery (PA). Typical trace of aorta (**a**) and pulmonary artery (**b**) in response to a stimulus of KPSS 80 mM for 3 min, and a dose response curve to different concentrations of PGF_2α_. (**c**) Average of the 3 min response to phenylephrine (PE) 1 µM (in PA (n = 15–52) and aorta (n = 9–16) of control, HF and HFHSu animal groups. (**d**) Dose response to PGF_2α_ (0.03–10 µM) in aorta of control, HF and HFHSu (n = 10 for all) animals. (**e**) Dose response to PGF_2α_ (0.03–30 µM) in PA of control (n = 21), HF (n = 12) and HFHSu (n = 7) animals. Values are presented as mean ± SD. One-way ANOVA with Dunnet’s Multiple Comparison test. *p < 0.05, **p < 0.01 and ****p < 0.0001 versus control animals; ^#^*p* < 0.05, ^##^*p* < 0.01, ^###^*p* < 0.001 and ^####^*p* < 0.0001, HF versus HFHSu animals.

Figure 1 shows a typical trace of the vascular contractile response to KPSS and cumulative doses of PGF_2α_ in the aorta (Fig. 1a) and PA (Fig. 1b). KPSS, an unspecific depolarizing stimulus, induced similar contractions in the PA and aorta of control or hypercaloric diet animals (data not shown). Contraction in response to 1 µM PE concentration showed no differences in either the PA or aorta in any of the disease states, prediabetes or early T2D, when compared with controls (Fig. 1c). We also tested 3 µM of PE in the aorta and there were no differences in vascular contractility among groups (data not shown). Because of the lack of differences among animals, and to further characterize the contractile properties of the arteries, we decided to use PGF_2α_, at different concentrations, as contracting agent. PGF_2α_ increased vascular aorta and PA contractility in a dose dependent manner (Fig. 1d, e). In the aorta (Fig. 1d) contraction to PGF_2α_ was significantly enhanced in the HF animals (contraction to PGF_2α_ 30 µM going from 75.3 ± 34.3% to 134.3 ± 54.4% of the KPSS contraction) while it did not changed in the HFHSu animals (contraction to PGF_2α_ 30 µM = 94.4 ± 44.1%). In the PA, contraction to PGF_2α_ 30 µM in control animals was 24.1 ± 16.2% of the KPSS response, while in HF animals it was enhanced to 32.0 ± 15.1% and diminished in the HFHSu (contraction to PGF_2α_ 30 µM = 10.6 ± 4.8%) (Fig. 1e).

**Figure 2:**
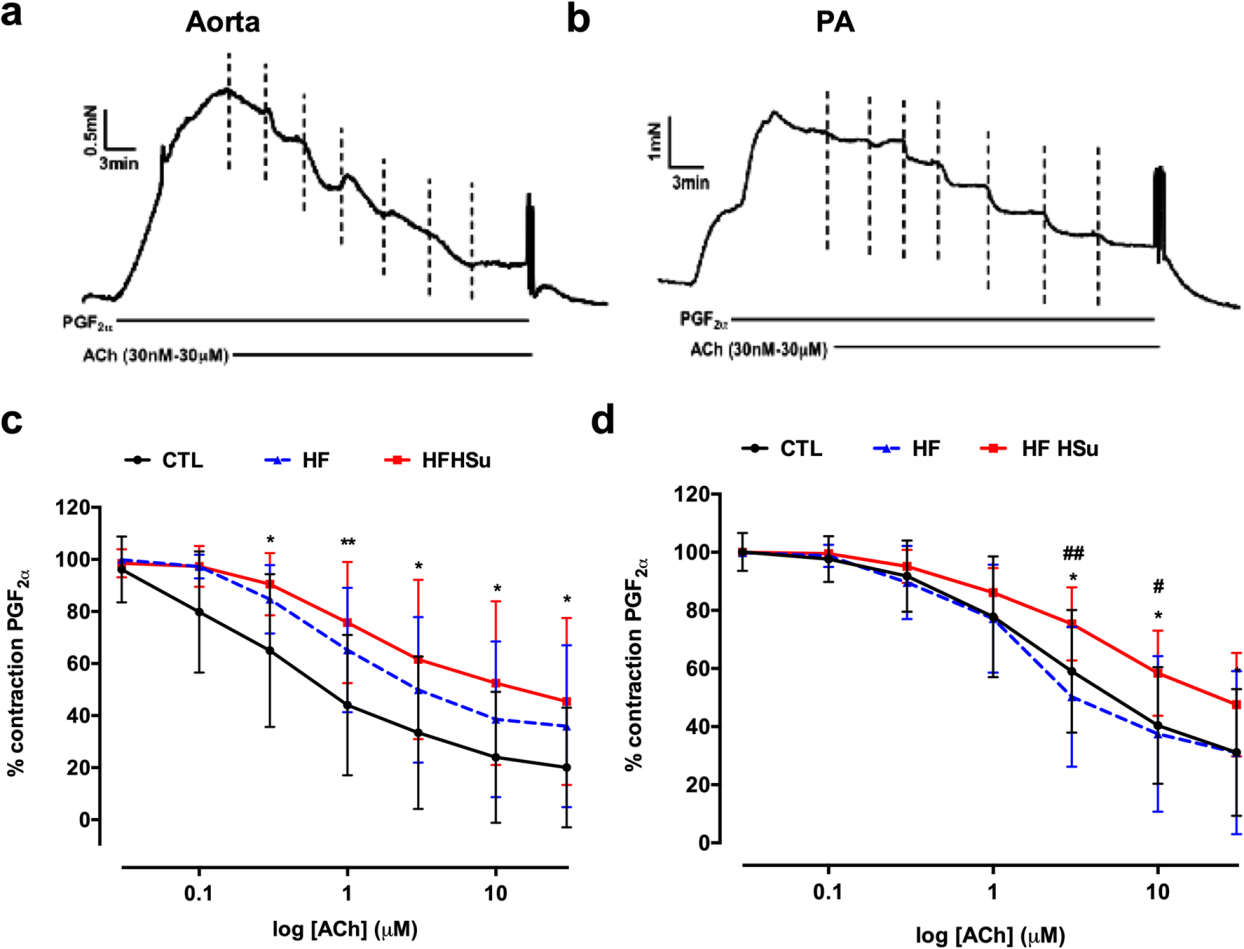
Effect of type 2 diabetes progression on endothelial function in aorta and pulmonary artery (PA). Typical trace of a dose response relaxation curve to different concentrations of ACh (0.03–30 µM) in the aorta (**a**) and pulmonary artery (**b**). Average responses in the aorta (**c**) and pulmonary artery (**d**) of the dose response relaxations in control (n = 10–14), HF (n = 10–11) and HFHSu (n = 10) animals. Values are presented as mean ± SD. Curves compared using two-way ANOVA with Dunnet’s Multiple Comparison test. *p < 0.05 and **p < 0.01 versus control animals; ^#^*p* < 0.05 and ^##^*p* < 0.01, HF versus HFHSu animals.

Figure 2 shows the typical changes in wall tension of the aorta (Fig. 2a) and PA (Fig. 2b) by cumulative doses of ACh (0.03–30 µM), after precontraction with a stable submaximal PGF_2α_ contraction, matched among arteries. This protocol was used as a test of endothelial integrity, as ACh releases NO from the endothelial layer of the arteries, and its relaxation can be used as an index of vascular integrity. In the aorta (Fig. 2c), both animals submitted to hypercaloric diets showed signs of endothelial damage (contraction after ACh 30 µM of 20.1 ± 23.0% in controls vs 35.9 ± 31.1% in the HF and 45.5 ± 32.1% in the HFHSu animals). In the PA (Fig. 2d) it was observed a diminished endothelium dependent vasorelaxation in the HFHSu animals (remaining contraction to ACh 30 µM 31.1 ± 21.8% in the controls vs 47.5 ± 17.8% in the HFHSu), which is a sign of endothelial damage, with no changes in the HF animals (with contraction after ACh 30 µM of 31.1 ± 28.0%). These data interestingly suggest that PA shows a greater resistance to endothelial damage than aorta. In PA, the endothelium of HF animals seemed to remains undamaged and endothelial dysfunction only appears in the HFHSu animal model. In aorta, endothelial damage is already notable in HF animals, although to a smaller extent than in HFHSu animals.

### Effect of diabetes progression on PGF_2α_ plasma levels and on the expression of PGF_2α_ receptors

**Figure 3:**
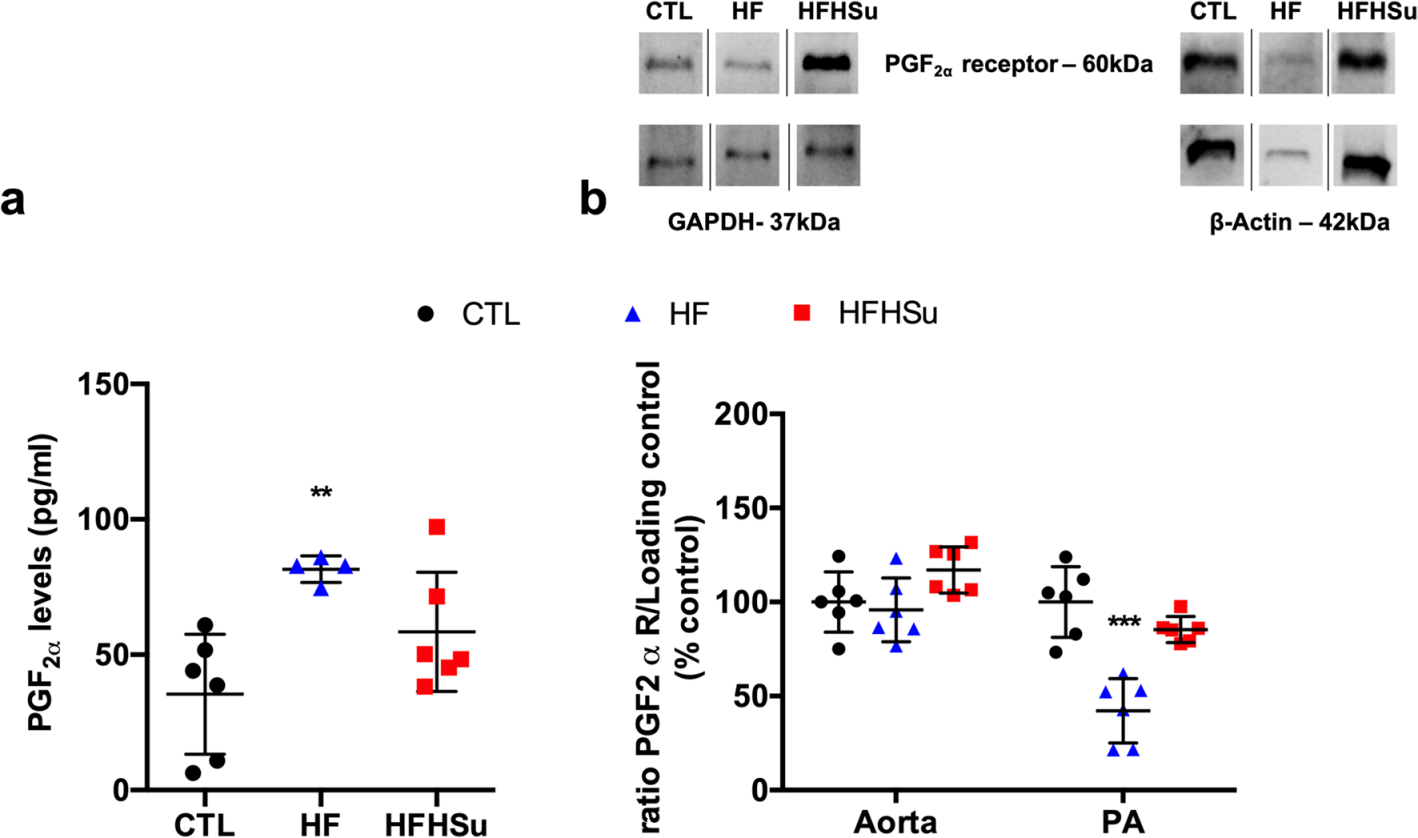
Effect of prediabetes and early phase type 2 diabetes on PGF2α levels in plasma and on the expression of PGF2α receptor. (**a**) PGF2α levels in plasma of control animals (n = 6) and animals submitted to HF (n = 4) or HFHSu (n = 6) diets. (**b**) Average relative expression of PGF2α receptor (60KDa) and the loading protein β-Actin (43 kDa) or GAPDH (37 kDa) in aorta and pulmonary artery (PA) of control animals (n = 6) and animals submitted to HF (n = 6) and HFHSu (n = 6) diets. Representative western blots for each protein studied are depicted above the respective graphs. For PGF2α expression, the representative bands were cropped due to the sample order in the membrane. For complete membrane image see Supplementary data. Values are presented as scatter plots ± SD; One-way ANOVA with Dunnet’s comparison test. **p < 0.01 and ***p < 0.001 versus control animals.

PGF_2α_ is a potent vasoconstrictor acting on the airways and on VSM cells to promote vasoconstriction and that has been implicated in the pathogenesis of pulmonary^25^ and cardiovascular disease^26^. Aiming to explore the alterations in the vasoconstrictor responses to PGF_2α_ in the aorta and PA produced by hypercaloric diets (Fig. 2c, d) herein we investigated the effects of diabetes progression on the levels of PGF_2α_ and on the expression of its receptors in aorta and PA (Fig. 3).

HF diet significantly increased PGF_2α_ levels in plasma by 130% in relation to control animals (PGF_2α_ control animals = 35.4 ± 22.1 pg/ml), while HFHSu diet only increased PGF_2α_ levels by 65% (Fig. 3a). In agreement with the increased levels of PGF_2α_ induced by HF diet, the expression of the PGF_2α_ receptor was decreased by 57.9% in the PA (Fig. 3b), although HFHSu diet did not modify the expression of PGF_2α_ receptor in this arterial wall. On the other side, in the aorta, HFHSu diet increased PGF_2α_ receptor expression by 17% while no alterations were observed in animals submitted to HF diet.

### Effect of diabetes progression on systemic and local nitric oxide levels and on the expression of eNOS and iNOS

**Figure 4:**
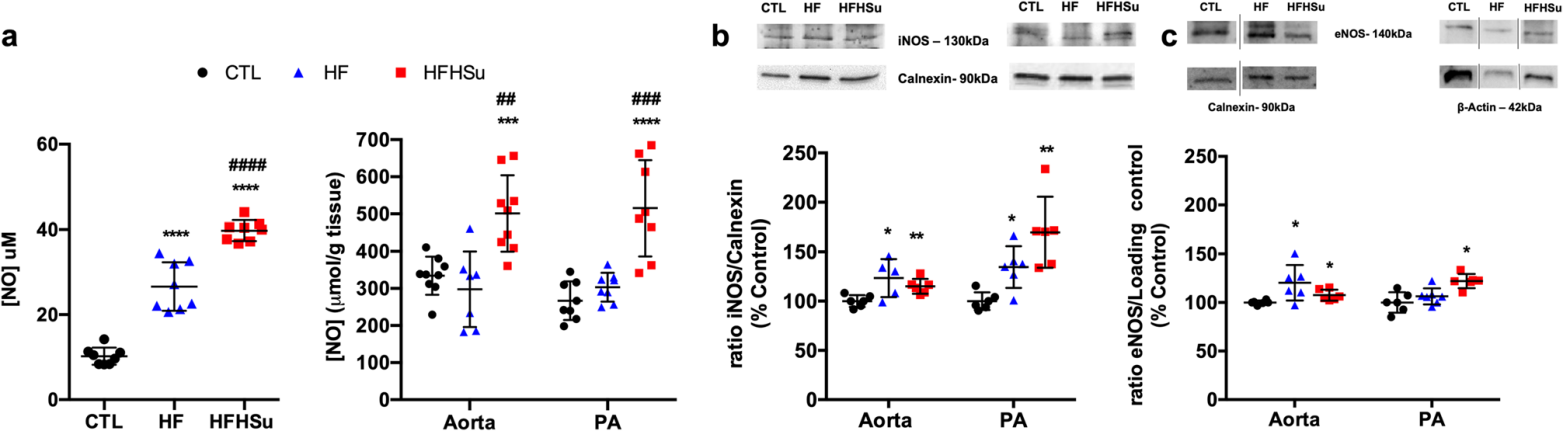
Effect of type 2 diabetes progression on systemic and local nitric oxide levels and on the expression of eNOS and iNOS. Nitric oxide levels on plasma (**a**, left panel) and on aorta and pulmonary artery (**a**, right panel); Average expression of (**b**) iNOS (130 kDa) and (**c**) eNOS (140 kDa), and the loading proteins, calnexin (90 kDa) and β-Actin (43 kDa) in the aorta and PA of control (n = 6), HF (n = 5–6) and HFHSu (n = 6) animals. Representative western blots for each protein studied are depicted above the respective graphs. For eNOS expression, the representative bands were cropped due to the sample order in the membrane. For complete membrane image see Supplementary data. Values are presented as scatter plots ± SD; One-way ANOVA with Dunnet’s comparison test. *p < 0.05, **p < 0.01, ***p < 0.001 and ****p < 0.0001 versus control animals; ^##^*p* < 0.01, ^###^*p* < 0.001 and ^####^*p* < 0.0001 comparing the groups submitted to the hypercaloric diets.

In Fig. 4 is represented the effect of diabetes progression, obtained by submitting the animals to different hypercaloric diets during different times of exposure, on the levels of NO in the plasma and in aorta and PA, and on the expression of eNOS and iNOS in both arteries.

Both hypercaloric diets increased NO plasma levels, with the HF diet promoting a significant increase of 160.3% and HFHSu inducing an even higher increase of 289% in the NO plasma levels in relation to controls (NO plasma levels in control animals of 10.2 ± 2.0 μM) (Fig. 4a left panel). When the NO levels were measured in both arteries, 3 weeks of exposure to HF diet were insufficient to promote significant alterations in the NO levels. However, HFHSu diet for 25 weeks increased NO levels by 50% in the aorta and by 93% in the PA when comparing with control animals (NO _control aorta_ = 334.2 ± 51.3 nmoles/g tissue; NO _control PA_ = 266.9 ± 52.0 nmoles/g tissue) (Fig. 4a right panel). In agreement with the increase in NO levels in the plasma and in the arteries, in the aorta both hypercaloric diets promoted an increase, of 23% and of 15%, for HF and HFHSu respectively, in iNOS. This increase of iNOS expression with both hypercaloric diets was also observed in the PA with an increase of 35% promoted by HF diet and of 70% promoted by the HFHSu diet (Fig. 4b). When eNOS expression was evaluated, it was observed an increase promoted by both hypercaloric diets in the aorta, 20% and 8% for HF diet and HFHSu diet, respectively, while only the HFHSu diet promoted an increase of 22% on eNOS expression in the PA in aorta (Fig. 4c).

### Effect of diabetes progression on inflammatory markers involved in endothelial dysfunction

**Figure 5:**
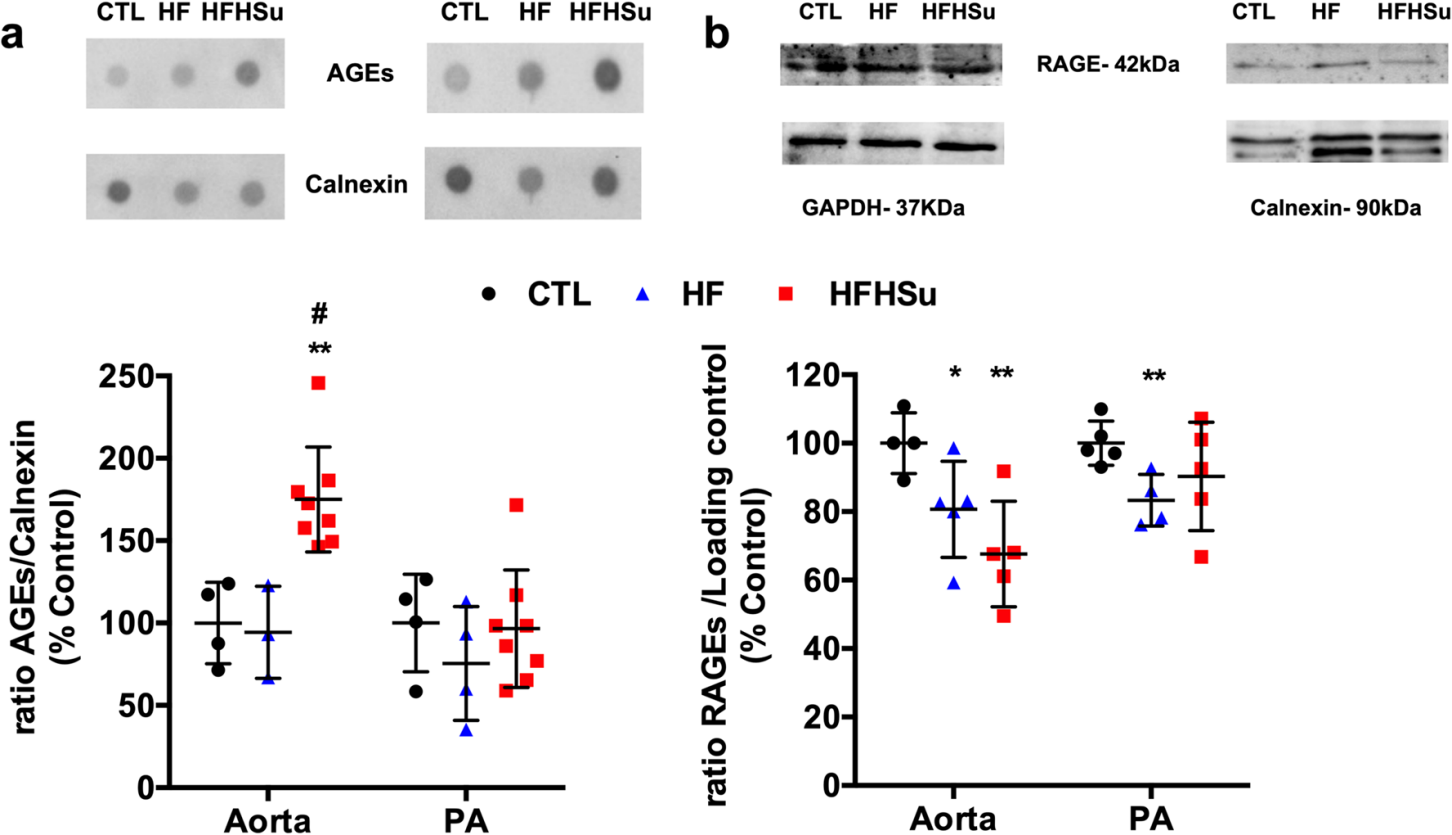
Effect of type 2 diabetes progression on AGEs levels and AGEs receptor expression. (**a**) Average relative levels of AGEs, and the loading protein Calnexin, on aorta and PA of control animals (n = 3–4) and animals submitted to HF (n = 3–4) or HFHSu (n = 6–8) diets. (**b**) Average relative expression of RAGE (42 kDa) and the loading protein used—Calnexin (90 kDa) or GAPDH (37 kDa)—in aorta and PA of control animals (n = 4–5) and animals submitted to HF (n = 4–5) and HFHSu (n = 4–5) diets. Representative western blots for each protein studied are depicted above the respective graphs. For complete membrane image see Supplementary data. Values are presented as scatter plots ± SD; One-way ANOVA with Dunnet’s comparison test. *p < 0.05 and **p < 0.01 versus control animals; ^#^*p* < 0.05 comparing the groups submitted to the hypercaloric diets.

Advanced glycation end products (AGEs) promote oxidative and inflammatory reactions in endothelial cells through the interaction with their receptor (RAGE)^27^. Herein, we observed that the levels of AGEs only increased in the aorta of the HFHSu animals by 75%, with no alterations on the PA (Fig. 5a). The expression of RAGE was significantly decreased by 19 and 32% in the aorta, respectively, with the HF and HFHSu diet, and by 15%, in the PA with the HF diet, with no alterations observed in the PA with the HFHSu diet (Fig. 5b).

**Figure 6:**
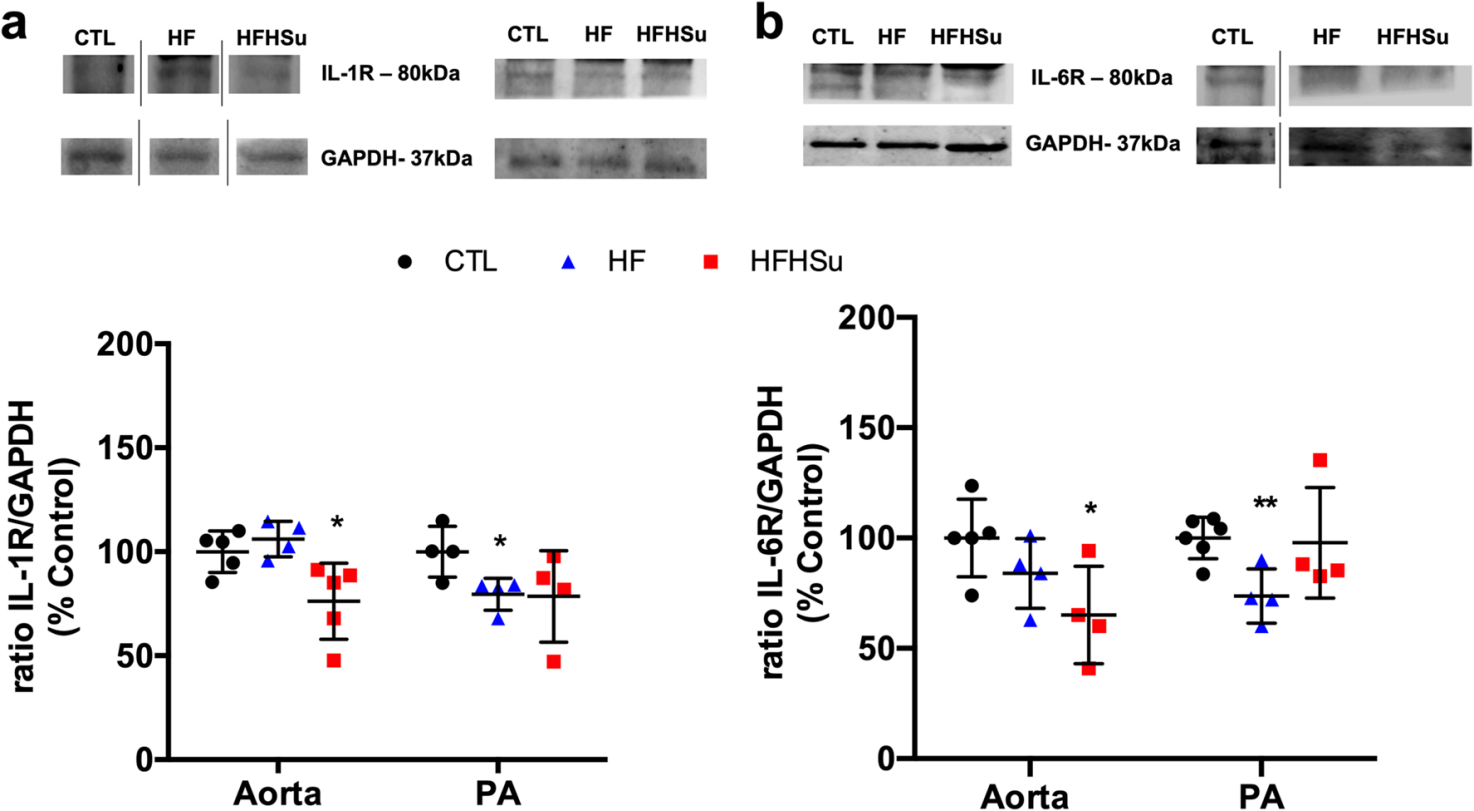
Effect of prediabetes and early phase type 2 diabetes on inflammatory markers involved in endothelial dysfunction. Average expression of (**a**) IL-1 receptor (IL-1R, 80 kDa) and (**b**) IL-6 receptor (IL-6R, 80 kDa) and the loading protein GAPDH (37 kDa) in aorta and pulmonary artery (PA) of control (n = 4–5), HF (n = 3–5) and HFHSu (n = 3–4) animals. Representative western blots for each protein studied are depicted above the respective graphs. Representative bands were cropped due to the sample order in the membrane. For complete membrane image see Supplementary data. Values are presented as scatter plots ± SD; One-way ANOVA with Dunnet’s comparison test. *p < 0.05 and **p < 0.01 versus control animals.

Inflammatory cytokines, such as interleukin-6 (IL-6) and 1 (IL-1), are considered as important contributors to endothelial dysfunction in T2D^10^. Herein we evaluated IL-1 (Fig. 6a) and IL-6 receptors expression (Fig. 6b) in both arteries. In the aorta, hypercaloric diets, and specifically HFHSu decreased IL-1R by 23 and IL-6R by 35%. In contrast, in the PA both HF and HFHSu diets decreased IL-1R by 20 and 21%, although the decrease in HFHSu was not significant (*p* = 0.058). Surprisingly, in the PA, only HF diet decreased the expression of IL-6R by 26%.

### Effect of diabetes progression on catalase expression

**Figure 7:**
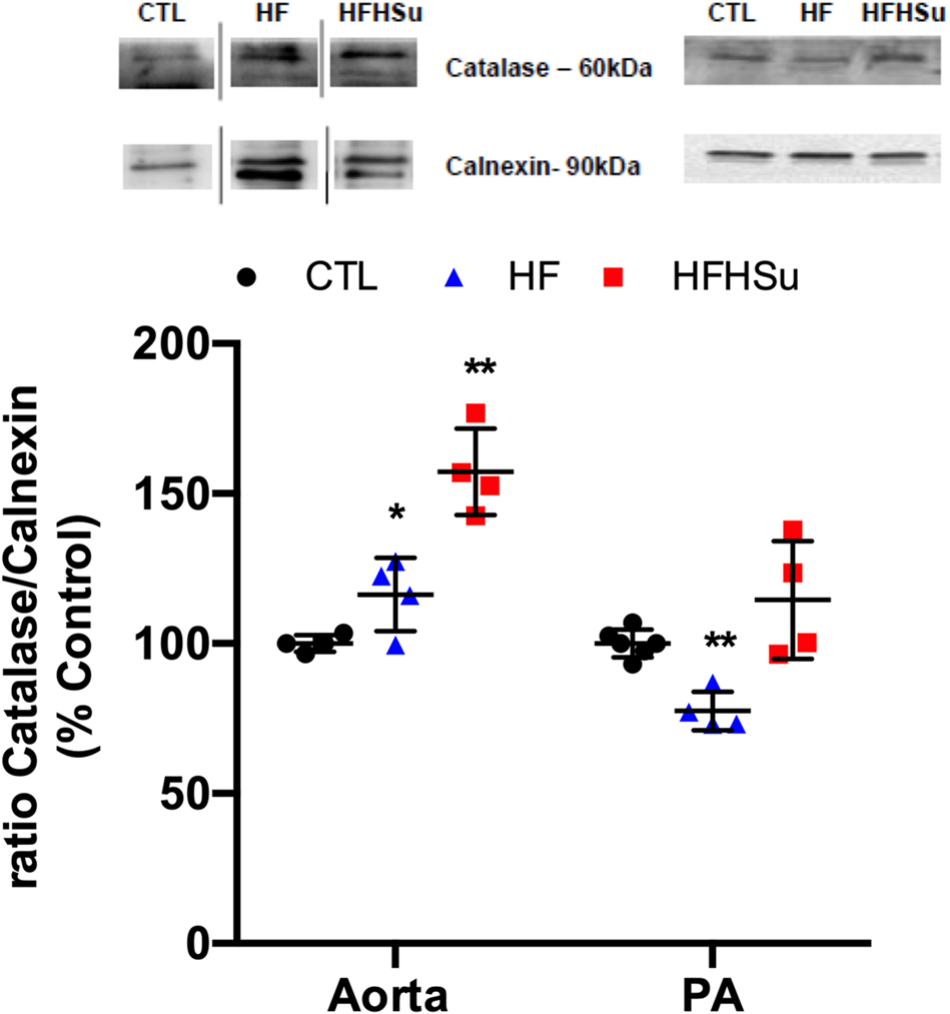
Effect of prediabetes and early phase type 2 diabetes on catalase expression. Average expression of catalase (60 kDa), and the loading protein calnexin (90 kDa) in the aorta and pulmonary artery (PA) of control (n = 4–6), HF (n = 4) and HFHSu (n = 4) animals. Representative western blots for each protein studied are depicted above the respective graphs. The representative bands were cropped due to the sample order in the membrane. For complete membrane image see Supplementary data. Values are presented as scatter plots ± SD; One-way ANOVA with Dunnet’s comparison test. *p < 0.05 and **p < 0.01 versus control animals.

Catalase is an antioxidant enzyme that, conjugated with antibodies to platelet-endothelial cell adhesion molecule-1, specifically binds to endothelium, quenching reactive oxygen species, such as H_2_O_2_, and alleviating vascular oxidative stress and inflammation^28^. We evaluated the expression of this enzyme and observed that HF and HFHSu diets promoted a significant increase by 16 and 57%, respectively, in the aorta. In contrast HF diet promoted a decrease by 23% in the expression of catalase in the PA, while no alterations were found for animals submitted to HFHSu diet (Fig. 7).

### Effect of diabetes progression on vascular mineralization

**Figure 8:**
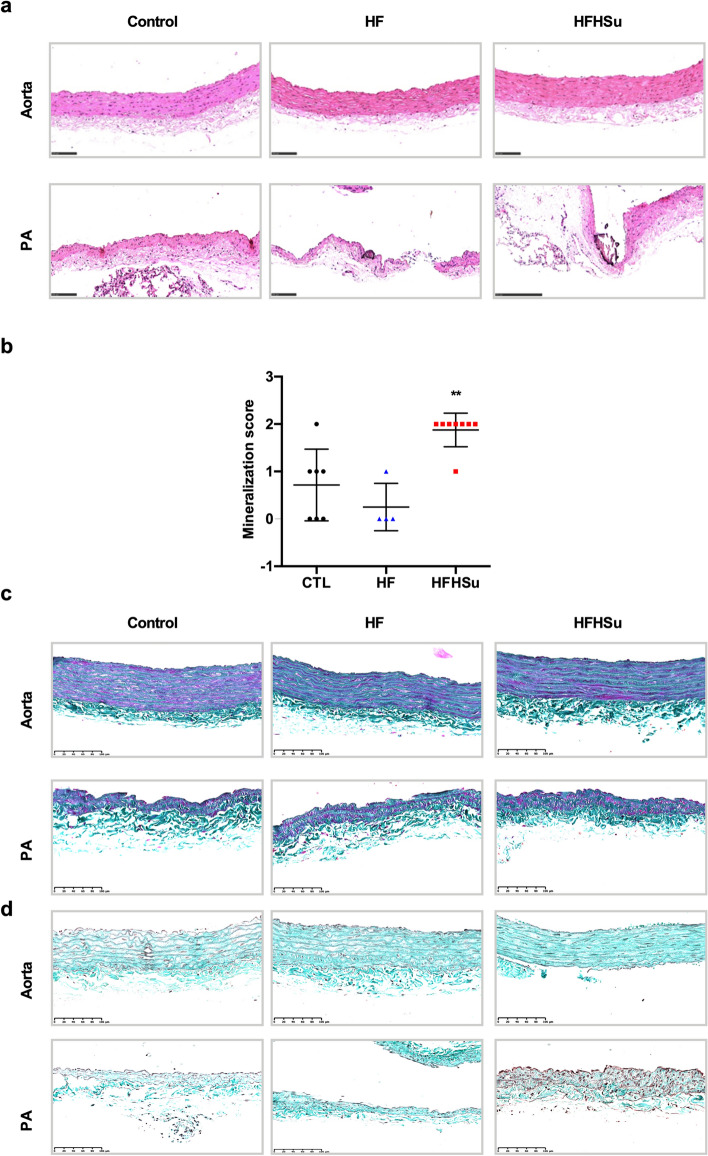
Effect of prediabetes and early phase type 2 diabetes on vascular mineralization. Histological analysis of the arteries stained with Hematoxylin and Eosin staining. No mineralization was observed on the aorta of the control, HF and HFHSu animals (**a**, top panel; left to right images, respectively). Mineralization was observed in the PA of various control and HF animals and in the PA of all the HFHSu animals (**a**, bottom panel; left to right images, respectively). (**b**) Vascular mineralization levels in PA of control (n = 7), HF (n = 4) and HFHSu (n = 8) animals. (**c**) Masson's trichrome staining for collagen evaluation. No alterations were observed on collagen in the aorta of the control, HF and HFHSu animals (c, top panel; left to right images, respectively) nor in the PA of the control, HF and HFHSu animals (c, bottom panel; left to right images, respectively). (**d**) Taenzer-Unna Orcein staining for elastin evaluation. No alterations were observed on elastin in the aorta of the control, HF and HFHSu animals (**d**, top panel; left to right images, respectively) nor in the PA of the control, HF and HFHSu animals (**d**, bottom panel; left to right images, respectively). Values are presented as mean ± SD. One-way ANOVA with Dunnet’s comparison test. **p < 0.01 versus control animals.

Vascular calcification is one of the major pathophysiological mechanisms on the basis of vascular disease, representing an independent risk factor for adverse outcomes. Through histological analysis of the arteries, stained with Hematoxylin and Eosin, it was possible to observe that no mineralization was present in the aorta arteries (Fig. 8a, top panel). However, mineralization was observed in the PA of some control and HF animals and in the PA of all HFHSu animals (Fig. 8a, bottom panel). Vascular mineralization was classified using a scoring system, from 0 to 3, that is based on the sum of the distribution and the layers/extent of deposition (Table 2). We observed that the score of mineralization was significantly higher in the HFHSu animals (mean score in control animals of 0.71 ± 0.76 and in the HFHSu of 1.88 ± 0.35) (Fig. 8b). Through Masson's trichrome and Taenzer-Unna Orcein staining, collagen and elastin were evaluated. However, no significant alterations were observed after the hypercaloric diets neither in collagen in the aorta (Fig. 8c, top panel) or in the PA (Fig. 8c, bottom panel) nor in the elastin in aorta (Fig. 8d, top panel) or PA (Fig. 8d, bottom panel).

**Table 2:** Effect of hypercaloric diets, high-fat (HF) and high fat-high sucrose (HFHSu), on pulmonary artery mineralization.

Experimental group	Pathology findings	Score (distribution)
CTL	Mineralization, intimal, focal	1
	No changes	0
	Mineralization, intimal, focal	1
	Mineralization, intimal and subintimal, multifocal	2
	No changes	0
	No changes	0
	Mineralization, intimal, focal	1
HF	No changes	0
	No changes	0
	No changes	0
	Mineralization, intimal, focal	1
HFHSu	Mineralization, intimal, subintimal and adventitia, multifocal	2
	Mineralization, intimal, multifocal	2
	Mineralization, intimal, multifocal	2
	Mineralization, intimal, multifocal	2
	Mineralization, intimal, multifocal	2
	Mineralization, intimal, multifocal	2
	Mineralization, intimal and media, multifocal	2
	Mineralization, intimal, focal	1

Vascular mineralization was classified using a scoring system, determined based on calcification in arterial cross section according to a 4-tier severity scale: 0, no calcification; 1, focal calcification spots; 2, partial calcification covering 20–80% of the arterial circumference; and 3, circumferential calcification.

## Discussion

Herein we found that T2D progression differently affects endothelial function and vascular contractility in the aorta and PA, with the contractile machinery being altered in the aorta and PA in prediabetes (HF animals) and in the PA of early stage T2D animals (HFHSu). Additionally, endothelial function was compromised in the aorta in both HF and HFHSu animals but only affected in the PA of HFHSu animals, suggesting that the PA is more resistant than aorta to endothelial dysfunction in early stages of disease progression. We found that PA and systemic endothelial dysfunction in diabetes progression in rats were associated with gradual alterations in the nitrergic system and inflammatory and oxidative pathways and we also described that PA dysfunction in T2D also involves endothelial wall mineralization.

Complex structural and functional changes occur in the arterial system with diabetes. We tested several vasoconstricting agents to study the influence of T2D progression on pulmonary and systemic vascular contraction. Phenylephrine did not change vascular contraction in any of the concentrations tested in both arteries, suggesting that sympathetic control of vascular contractility is not altered in the diabetic disease states evaluated in the present study (Fig. 1c). This contrasts with the findings that patients with T2D exhibited heightened norepinephrine-mediated vasoconstriction due to an increased alpha-adrenoreceptors for their level of systemic sympathetic nervous system activity^29^. However, these differences might reflect the low glucose levels of our animal models (Table 1) in comparison with T2D patients as well as the stage of disease progression. PGF_2α_ was also used, at different concentrations, to study vascular contractility and as contracting agent to study endothelial function. PGF2α is known to promote resistance artery constriction through prostaglandin F2α receptor in smooth muscle cells, leading eventually to increased blood pressure^26^. Herein, PGF_2α_ increased vascular PA contractility in a dose dependent manner in HF animals and diminished in a dose dependent manner in the HFHSu. In the aorta contraction to PGF_2α_ was significantly enhanced in the HF animals while slightly increased, although not significantly, in the HFHSu animals. This increase in vascular contractility in HF animals, both in aorta and PA, is in accordance with findings showing that elevated glucose increases contractile responses in vascular smooth muscle cells^30,31^ and with the increased plasma PGF_2α_ levels described herein (Fig. 3a) that can contribute to the increased contractility. In the PA, this was accompanied by a decrease in the expression of PGF_2α_ receptors (Fig. 3b), suggesting a compensation of the system to the increased PGF_2α_ plasma levels however, this compensation might be not enough to maintain vascular PA contractility in HF animals. In fact, we cannot rule out that alterations in other constrictor factors, as endothelin-1, might contribute to the increased PGF_2α_ vasoconstrictor responses in both aorta and PA. Vascular contractility to PGF_2α_ in HFHSu animals was lower in the PA in comparison with control animals, being similar in the aorta (Fig. 1d–e). These differences from HF to HFHSu animals might reflect adaptations of arterial walls to maintain vascular integrity, as seen by the unaltered PGF_2α_ levels in blood (Fig. 3a) and in the levels of their receptors in the arteries (Fig. 3b). From the several factors contributing to altered vascular contractility responses, one is arterial calcification that leads to increased mechanical vessel rigidity and stiffness^32^. Herein, we described that the PA of HFHSu animals exhibit increased artery mineralization with the development of intima and multifocal thickening (Fig. 6), changes not seen in the aorta. These differences in mineralization between the two arteries suggest that this is one of the mechanisms contributing to the changes in the vascular PA mechanical properties in HFHSu that is not occurring in the aorta.

Impaired endothelium-dependent vasodilation has been described in several vascular beds of different animal models of diabetes and in diabetic patients, including the aorta (for a review see Avogaro et al., 2011)^17^, however how T2D diabetes progression affects endothelium-dependent vasodilation and the endothelium-derived relaxing factors involved in PA dysfunction are less known. In the PA, ACh-dependent vasorelaxation, after precontraction with a stable submaximal PGF_2α_ concentration, did not change with HF diet, but decreased in HFHSu animals (Fig. 2d) while in the aorta, ACh-induced vasodilation gradually decreases with disease progression, with HFHSu animals exhibiting a lower vasodilatation than HF than the controls (Fig. 2d), which suggest that these two vascular beds respond differently to hypercaloric stimulus being PA more resistant to endothelial dysfunction. One of the major mechanisms that have been shown to be involved in systemic endothelial dysfunction in diabetes is an impairment of signal transduction or substrate availability of NO^33,34^, which contrasts with our data that shows increased NO levels in plasma and in HFHSu aorta samples (Fig. 4a).The source of these increased NO levels is the augmented expression of iNOS and eNOS (Fig. 4b–c), reflecting an inflammatory-induced NO production. Similar to iNOS, some authors found that eNOS by binding to calcium-calmodulin at basal Ca^2+^ levels in cytokine-treated endothelial cells, enhances its basal activity and is activated independently of increased [Ca^2+^]i^35^. So, not only iNOS- but also eNOS-derived NO becomes prominent under inflammatory conditions and in fact, it has been seen that eNOS can be activated by IL-6^36^. The role of endothelium-derived NO in the vascular function in the PA is poorly understood. Evidences suggest that NO is at least partially responsible for resting pulmonary vasorelaxation^37^ and some studies suggest that a deregulation on NO production, associated with either an increase or decrease on eNOS expression, is present in pulmonary hypertension^38–40^. In agreement with the presence of a decreased ACh-dependent vasorelaxation in HFHSu animals in the PA showed herein (Fig. 2c), we observe increased levels of NO in this arterial wall (Fig. 4a right panel), that were due to increased iNOS and eNOS expression (Fig. 4b–c), suggesting that these NO levels correspond to inflammatory NO. Additionally, while iNOS expression increased with disease progression, eNOS alterations were not found in HF animals (Fig. 4c), suggesting that this might be one of the mechanisms involved in the protection of PA endothelial function in an initial stage of the disease.

Another mechanism postulated to be involved in the link between diabetes and vascular complications is the increased formation of AGEs. The binding of AGEs to their receptors has been shown to enhance oxidant stress and induce increased vascular permeability and reduced NO-dependent vasodilation^8^. Herein, we observed that AGEs levels only increase in the aorta in HFHSu animals with no alterations promoted by 3 weeks of HF in both arteries or by HFHSu in the PA (Fig. 5a). Additionally, we described that the expression of AGE receptor, progressively diminished in the aorta with disease development being also significantly decreased in the PA with the HF diet (Fig. 5b). Altogether these results indicate a progressive inflammatory state. The increase in circulating and tissue AGEs levels was already demonstrated in both animal and human studies of diabetes^41–43^. In fact, in animal models of diabetes, AGEs levels increase with the progression of the disease, reflected by augmented levels in kidneys, skin, and vascular tissue^43^. Furthermore, it was already demonstrated that the increase in serum AGEs is correlated with the degree of impairment of endothelium-dependent and -independent vasodilation in patients with T2D^8^. Although our data, in the aorta, is in agreement with the literature, no correlation between AGE levels, RAGE expression and disease progression was seen in the PA, suggesting compensatory mechanisms during more prolonged exposure to hypercaloric diets (HFHSu vs HF) or the involvement of other inflammatory mediators. Another two inflammatory mediators that are deeply involved in endothelial dysfunction are IL-1 and IL-6^10^_,_ being the increase in these cytokines levels well reported in response to hypercaloric diets in animals and in T2D patients^44,45^. In agreement, we found that in general, IL-1R and IL-6R expression in the aorta and PA decrease progressively with disease development (Fig. 6), suggesting higher levels of the corresponding cytokines. Confirming the inflammatory involvement in endothelial dysfunction, IL-1R and IL-6R expression follow the same pattern of expression than RAGE in both aorta and PA, this being an expected result as it is well described that AGE-RAGE interaction modulates the generation of pro-inflammatory molecules and pathways in endothelial cells in several diseases, including diabetes^46,47^. Apart from the modulation of inflammation, AGE-RAGE interaction also modulates the generation of reactive oxygen species^46,48^ these last mediators also contributing for endothelial dysfunction^7,8^. In agreement with this, we found that the expression of catalase, an antioxidant enzyme that alleviates vascular oxidative stress and inflammation^28^ increase gradually with disease progression in the aorta, suggesting a compensatory mechanism to overcome the increase in reactive oxygen species produced by hypercaloric diets. In contrast in the PA, there was a decrease in catalase expression with the HF diet (Fig. 7), which together with decreased AGE-RAGE signalling, might suggest that ROS levels are diminished in this arterial wall being this another mechanism contributing to maintain PA wall integrity in early phases of diabetes. The absence of significant molecular alterations in the PA in a more advanced disease state, in the HFHSu animals, together with the existence of a low contractility (Fig. 1) and low relaxation (Fig. 2) suggests that other major mechanisms as the vascular wall calcification (Fig. 8) play a more important role in endothelial dysfunction.

We conclude that T2D progression differently affects endothelial function and vascular contractility in the aorta and PA, being the PA more resistant to endothelial dysfunction than the aorta. Altogether our findings show that the balance between the nitrergic system, inflammatory signals and reactive oxygen species are crucial to understand and discriminate the differences between these two arteries during metabolic disease progression. This can lead to significant advances in both preventative and therapeutic treatments in early stages of metabolic disease associated with PA endothelial dysfunction.

## Supplementary Information

Supplementary Information 1.

## Data Availability

The data that support the findings of this study are available from the corresponding author upon request.

## Abbreviations

Ach: Acetylcholine
AGEs: Advanced glycation end products
eNOS: Endothelial NOS
HF: High-fat
HFHSu: High fat-high sucrose
IL-1R: Interleukin 1 receptor
IL-6R: Interleukin 6 receptor
iNOS: Inducible NOS
ITT: Insulin tolerance test
NFκB: Nuclear factor kappa-light-chain-enhancer of activated B cells
NO: Nitric oxide
OGTT: Oral glucose tolerance test
PA: Pulmonary artery
PAH: Pulmonary arterial hypertension
PE: Phenylephrine
PGF_2α_: Prostaglandin F_2α_
RAGE: Receptor for advanced glycation end products
SD: Standard deviation
T2D: Type 2 diabetes
TNFα: Tumour necrosis factor alpha
VSM: Vascular smooth muscle
