# Supplementary material for: Type 2 diabetes progression differently affects endothelial function and vascular contractility in the aorta and the pulmonary artery
Source: Sci Rep. 2021 Mar 15;11:6052. doi: 10.1038/s41598-021-85606-7 (PMC7960698; doi:10.1038/s41598-021-85606-7)

Type 2 diabetes progression differently affects endothelial function and vascular contractility in the aorta and the pulmonary artery

Bernardete F. Melo[1,*], Jesus Prieto-Lloret[1*], Marlene D. Cabral[1], Fatima O. Martins[1], Inês B. Martins[1], Joana F. Sacramento[1], Pedro Ruivo[2], Tânia Carvalho[2] and Silvia V. Conde[1]

Supplementary Data

Table S1 – Composition of Standard diet

**Composition**	**%**
**Proximate Analysis**	
Moisture	10.00
Crude Oil	4.16
Crude Protein	21.86
Crude Fibre	4.33
Ash	7.89
Nytrogen Free Extract	51.24
**Amino Acids**	
Arginine	1.39
Histidine	0.54
Isoleucine	0.96
Leucine	1.81
Lysine	1.30
Phenylalanine	1.20
Tyrosine	0.85
Alanine	0.27
Aspartic Acid	1.34
Glutamic Acid	4.30
Proline	1.53
Serine	0.98
**Fatty acids**	
Lauric	0.05
Myristic	0.17
Palmitic	0.37
Stearic	0.10
Myristoleic	0.01
Palmitoleic	0.09
Oleic	1.00
Linoleic	1.25
Linolenic	0.17
Arachidonic	0.12
**Fiber and Carbohydrates**	
Total Dietary Fibre	15.70
Pectin	1.47
Hemicellulose	9.33
Cellulose	4.04
Lignin	1.50
Starch	33.61
Sugar	5.84
**Energy**	
Gross Energy	15.10 MJ/kg
Digestible Energy	12.27 MJ/kg

Metabolizable energy	11.24 MJ/kg
Atwater Fuel Energy (AFE)	13.79 MJ/kg
AFE from Oil	11.35
AFE from Protein	26.51
AFE from Carbohydrate	62.13
**Minerals**	
Calcium	1.24
Phosphorus	0.80
Potassium	0.78
Magnesium	0.28
Sodium	0.24
Chloride	0.36
Fluorine	8.53 mg/kg
Iron	161.01 mg/kg
Zinc	46.90 mg/kg
Manganese	101.71 mg/kg
**Vitamins**	
Vitamin A	42522.66 IU/kg
Vitamin D-3 (added)	4369.41 IU/kg
Vitamin E	171.70 IU/kg
Vitamin K	40.72 mg/kg
Thiamin	49.77 mg/kg
Riboflavin	37.74 mg/kg

Table S2 – Composition of 60% lipid-rich diet

Composition	%
**Protein**	**23.1**
Arginine	0.90
Histidine	0.67
Isoleucine	1.24
Leucine	2.24
Lysine	1.88
Phenylalanine	1.24
Tyrosine	1.31
Threonine	1.0
Valine	1.47
Aspartic Acid	1.66
Glutamic Acid	5.28
Proline	3.04
Serine	1.43
**Fat**	**34.9**
Cholesterol	301 ppm
Linoleic Acid	4.70
Linolenic Acid	0.39
Arachidonic Acid	0.06
Omega-3 Fatty Acids	0.39
Total Saturated Fatty A	13.68
Total Monounsaturated Fatty Acids	14.00
Polyunsaturated Fatty Acids	5.15
**Fiber (max)**	**6.5**
**Carbohydrates**	**25.9**
**Energy**	**5.10 kcal/g**
**From:**	**%**
Protein	18.1
Fat	61.6
**Minerals**	
Calcium	0.79
Phosphorus	0.59
Potassium	0.77
Magnesium	0.07
Sodium	0.15
Chloride	0.25
Fluorine	1.2 ppm
Iron	65 ppm
Zinc	46 ppm

Manganese	76 ppm
**Vitamins**	
Vitamin A	5.2 IU/g
Vitamin D-3 (added)	1.3 IU/g
Vitamin E	67.2 IU/kg
Vitamin K	0.65 ppm
Thiamin	6.2 ppm
Riboflavin	8.7 ppm
Niacin	39 ppm
Pantothenic Acid	21 ppm

Supplementary Data - Representative Western Blot images

Figure 3 b

$PGF_{2\alpha}$ receptor

Aorta

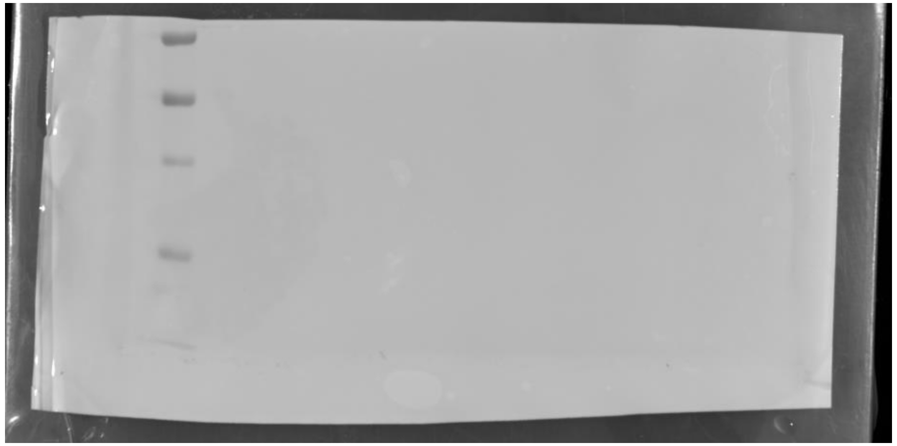

Representative bands cropped for the manuscript

CTL HF HFHSu

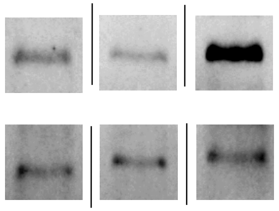

$PGF_{2\alpha}$ receptor - 60KDa

GAPDH- 37KDa

$PGF_{2\alpha}$ receptor
60KDa

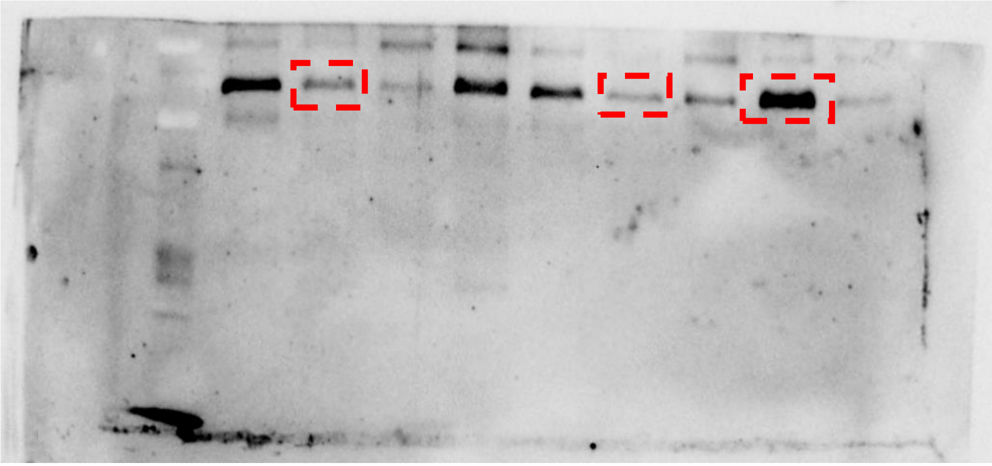

CTL HF HF HFSU

GAPDH
37KDa

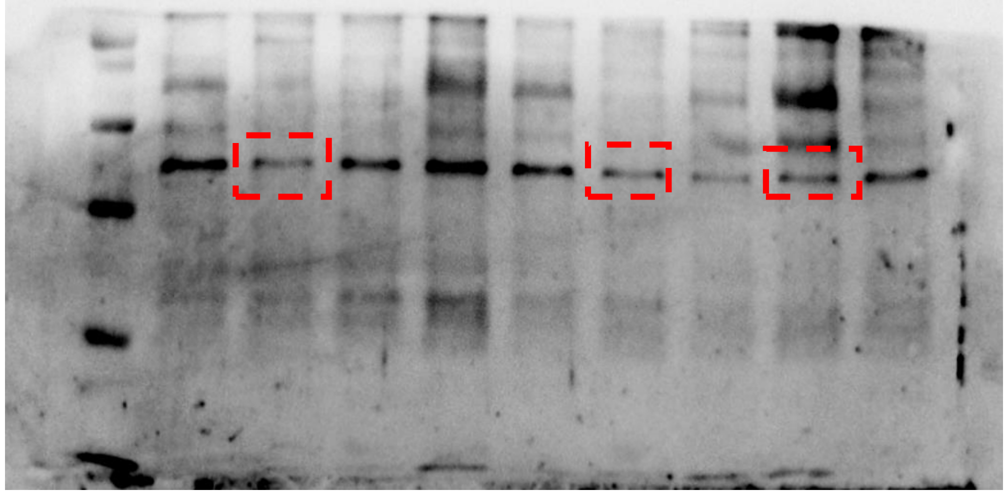

Figure 3 b

$PGF_{2\alpha}$ receptor

Pulmonary Artery

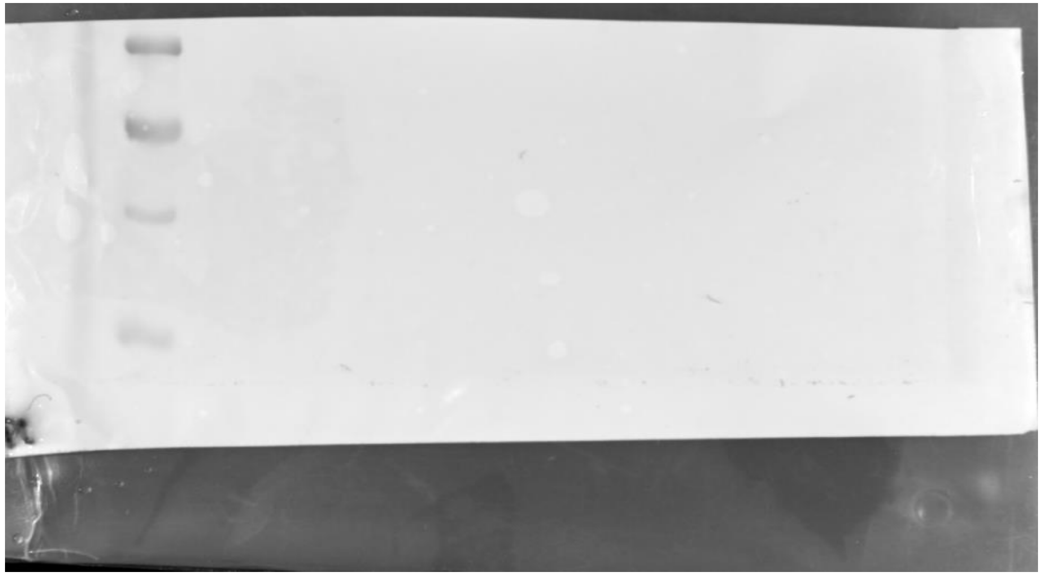

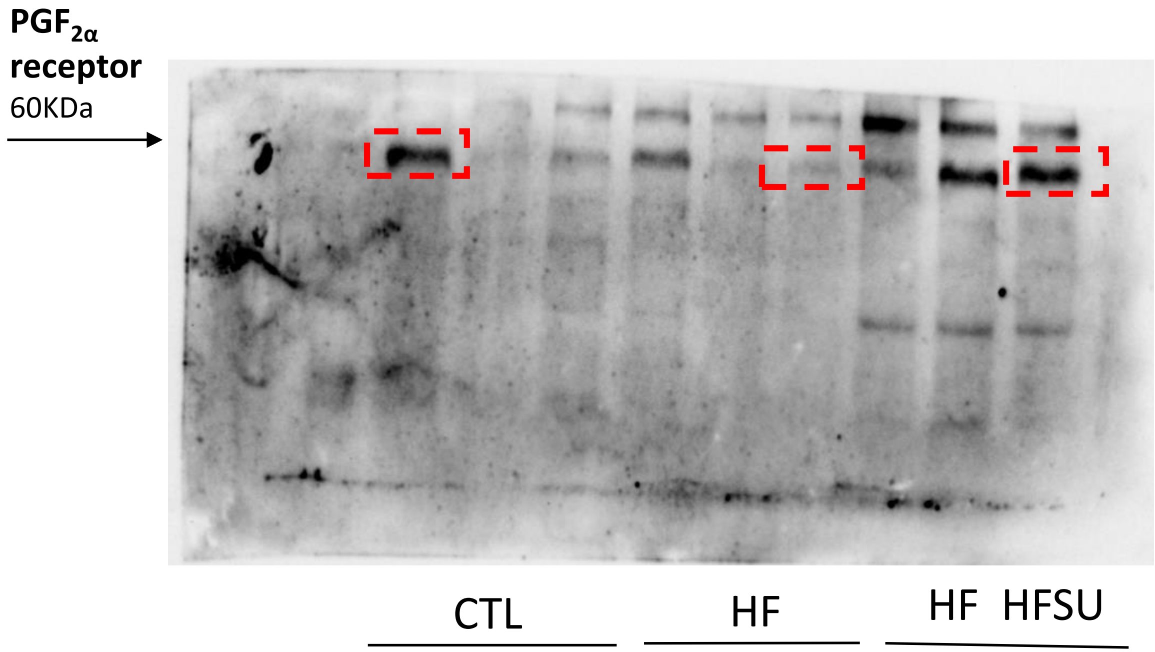

Representative bands cropped for the manuscript

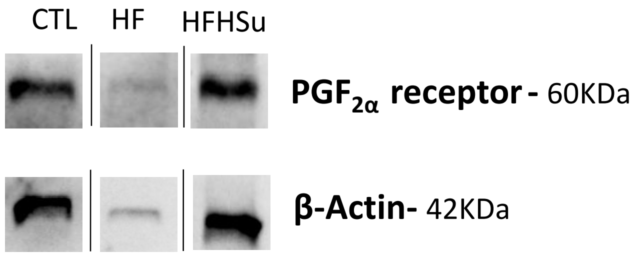

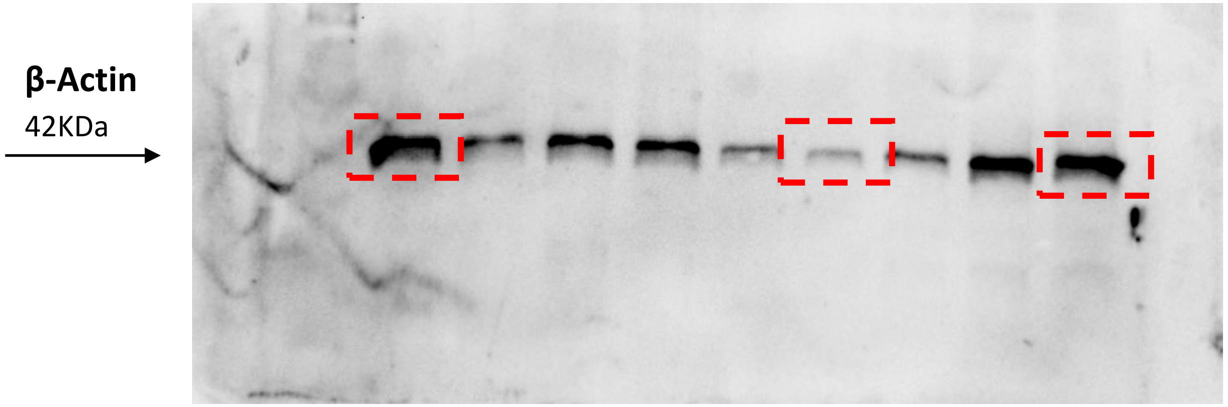

Figure 4 b

iNOS

Aorta

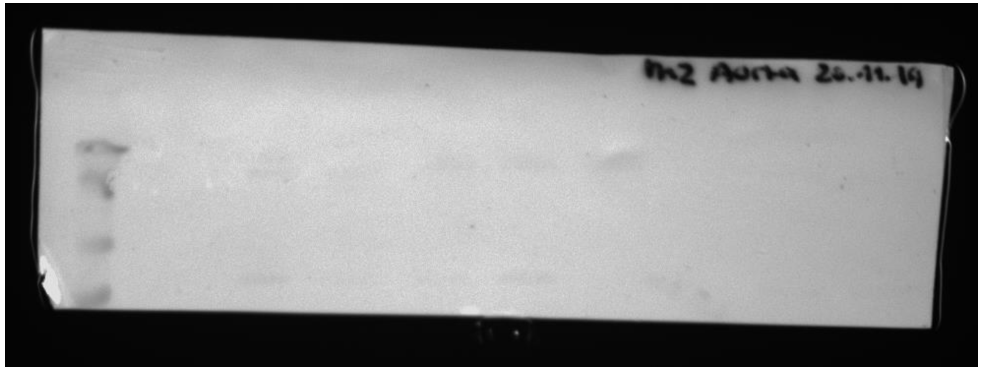

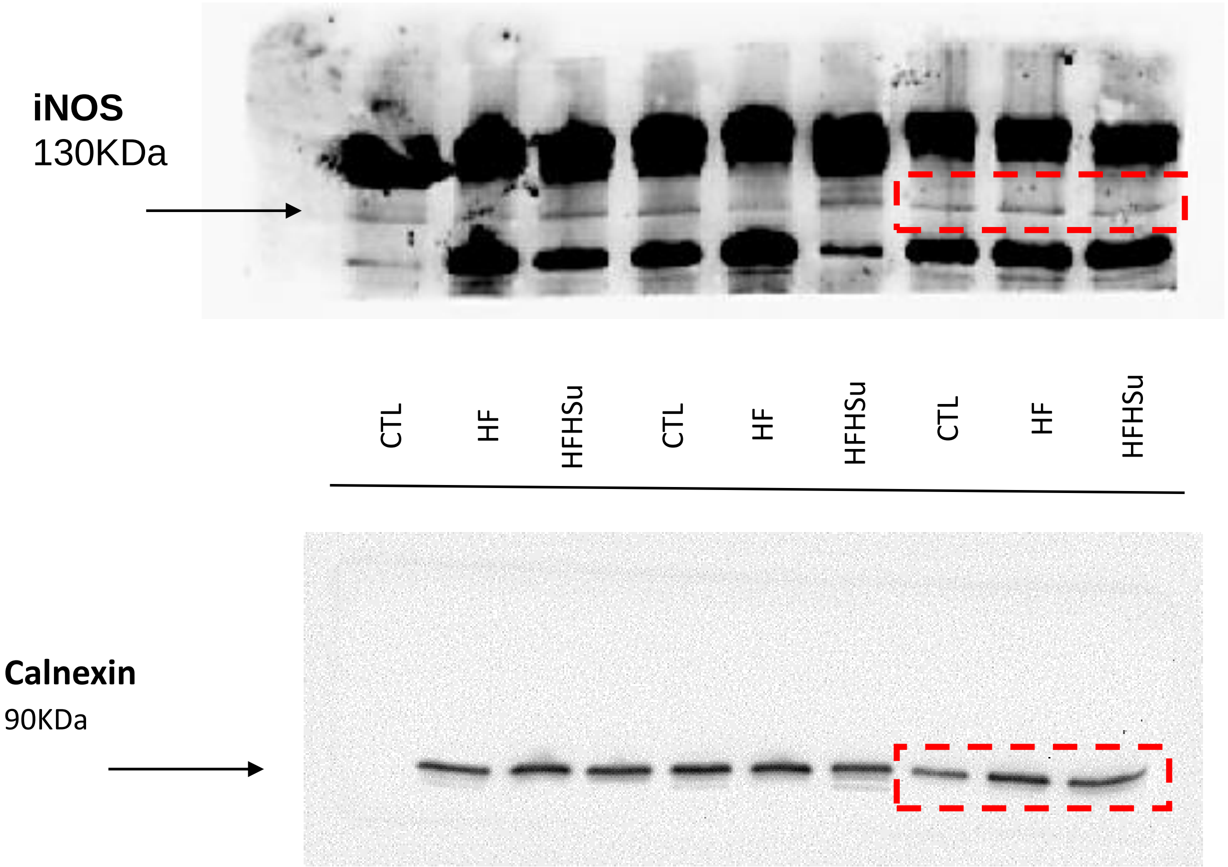

Figure 4 b

iNOS

Pulmonary artery

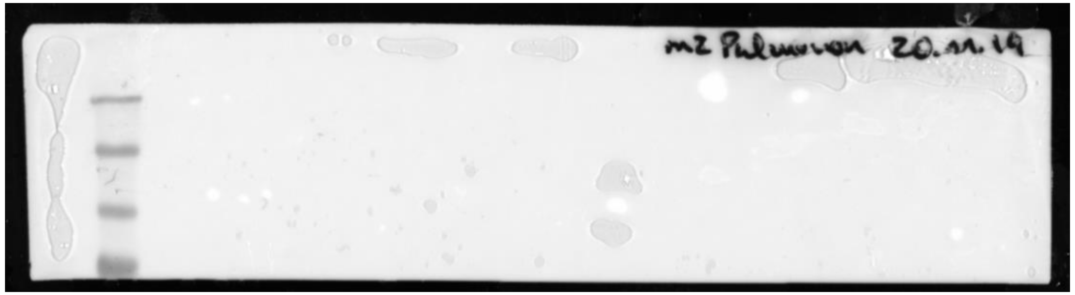

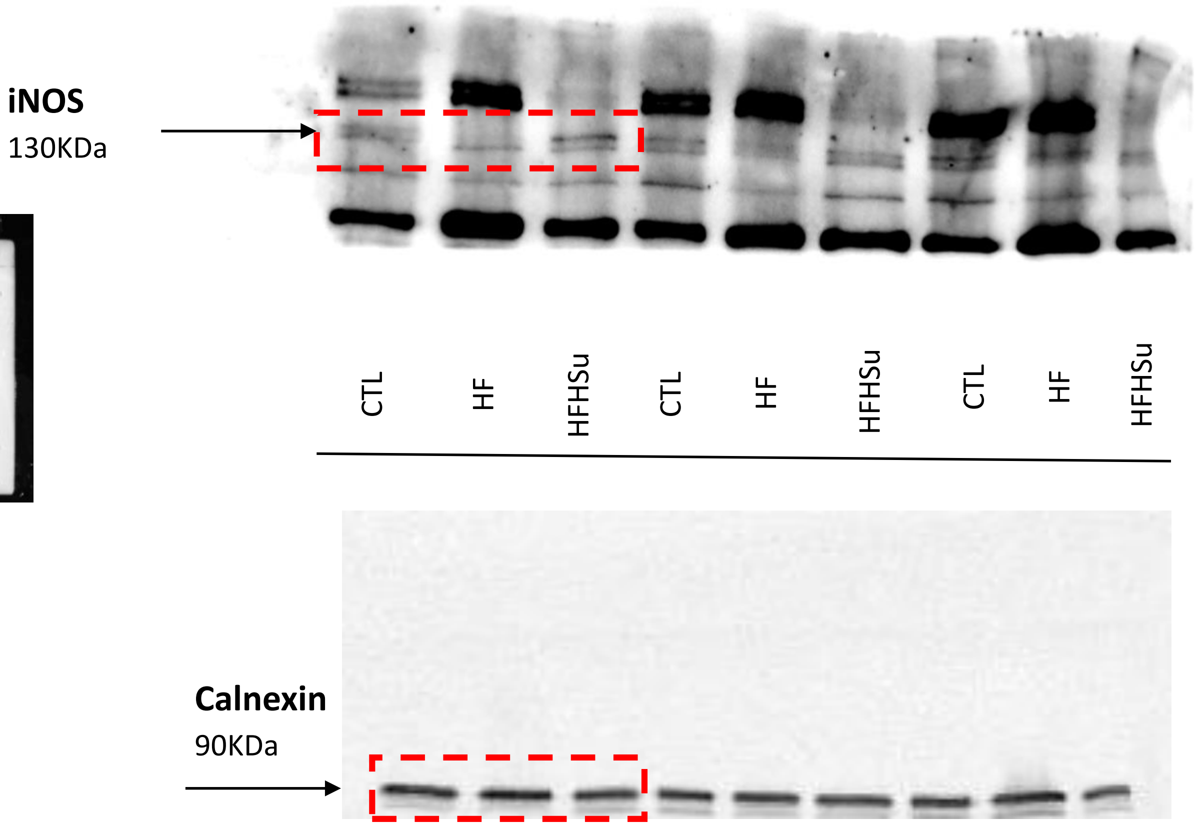

Figure 4 c

eNOS

Aorta

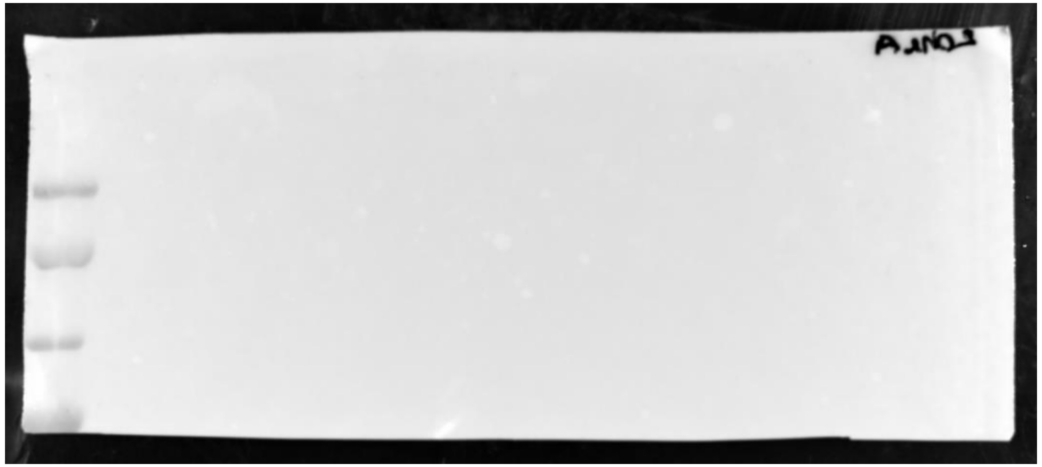

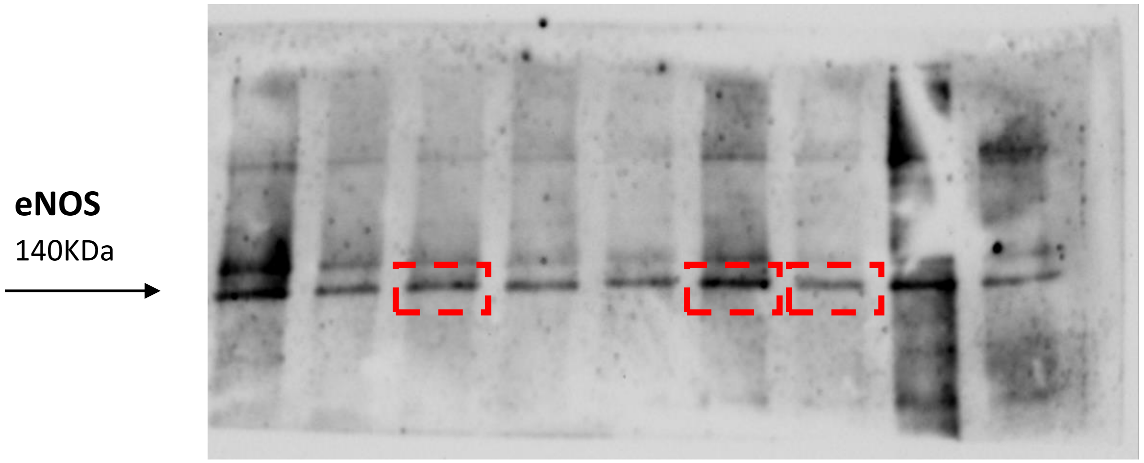

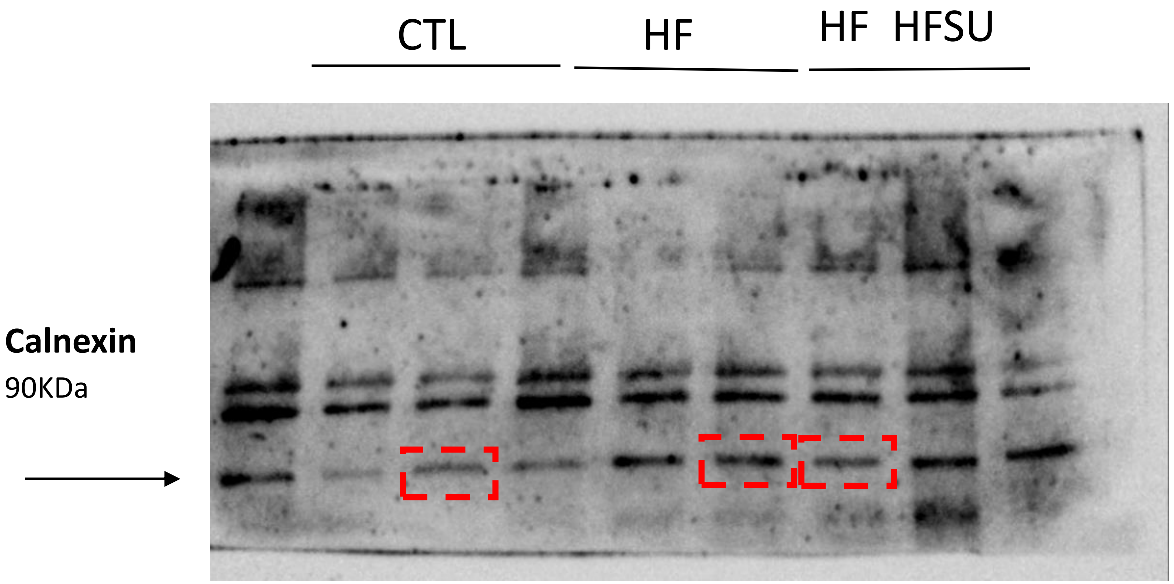

Representative bands cropped for the manuscript

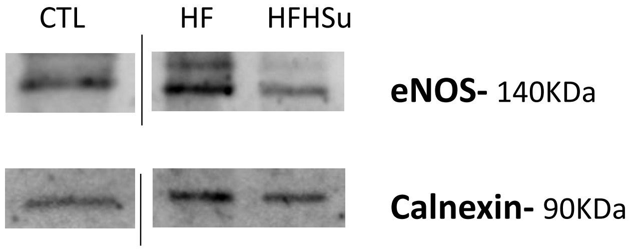

Figure 4 c

eNOS

Pulmonary Artery

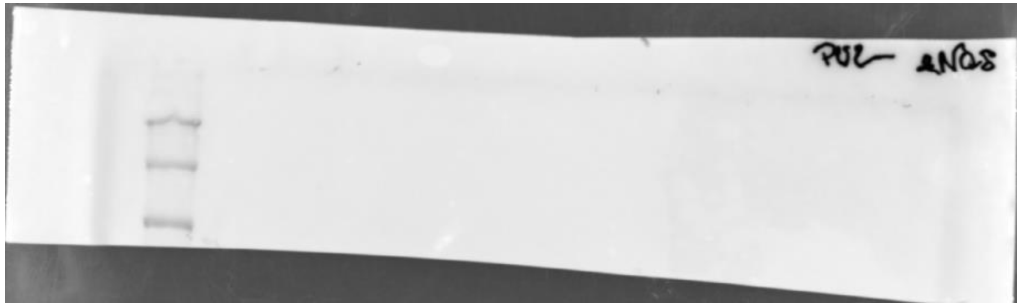

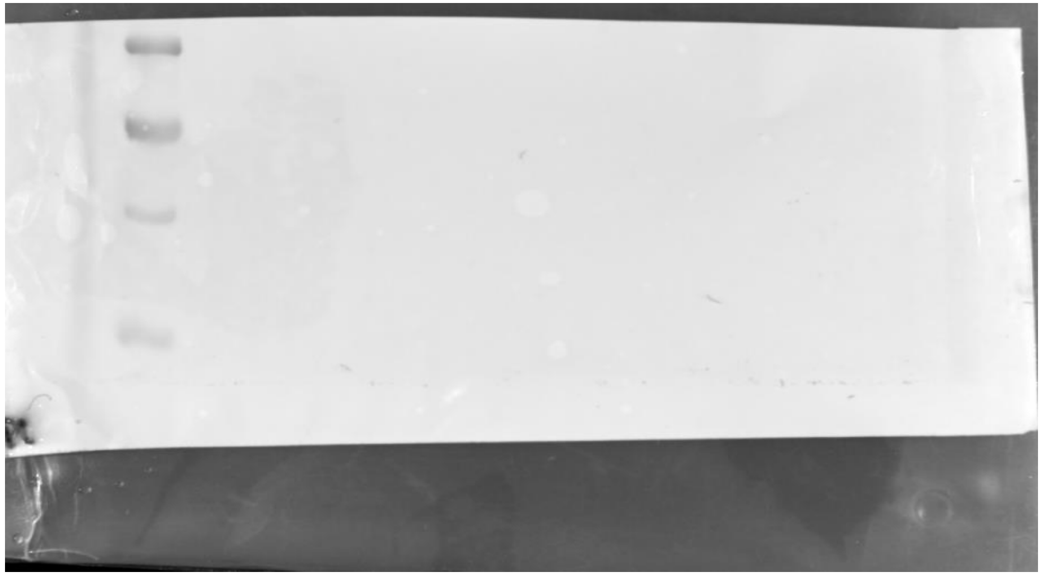

Representative bands cropped for the manuscript

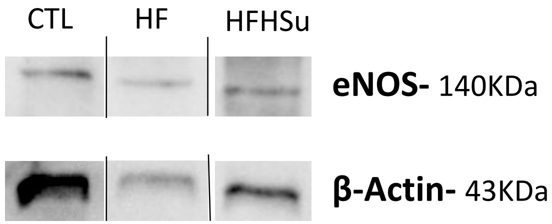

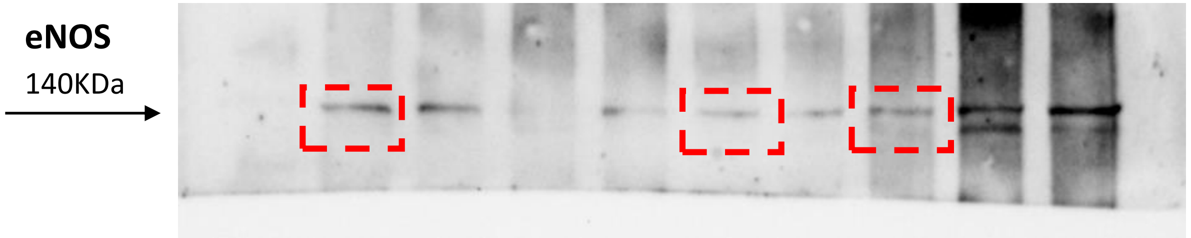

CTL HF HF HFSU

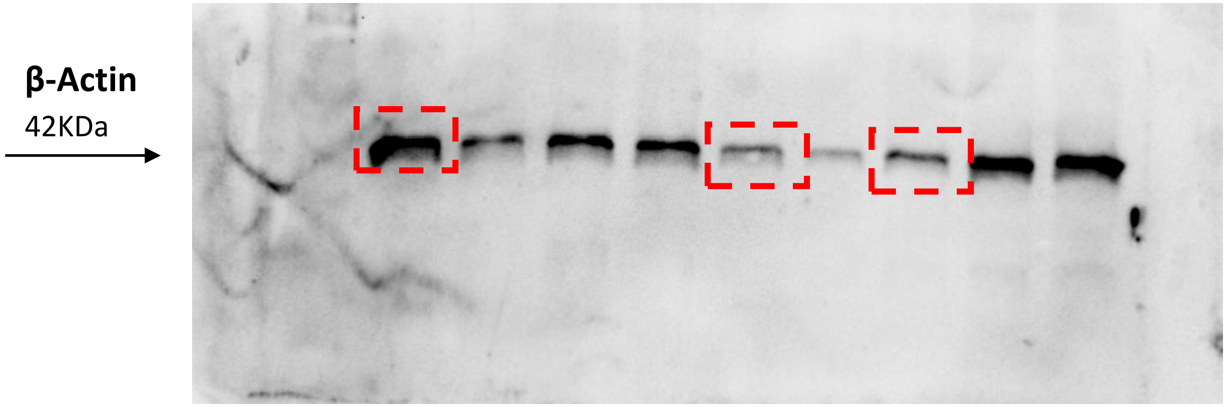

Figure 5 a

AGEs [N^{ε}-(carboxyethyl) lysine (CEL)]

Aorta and Pulmonary artery

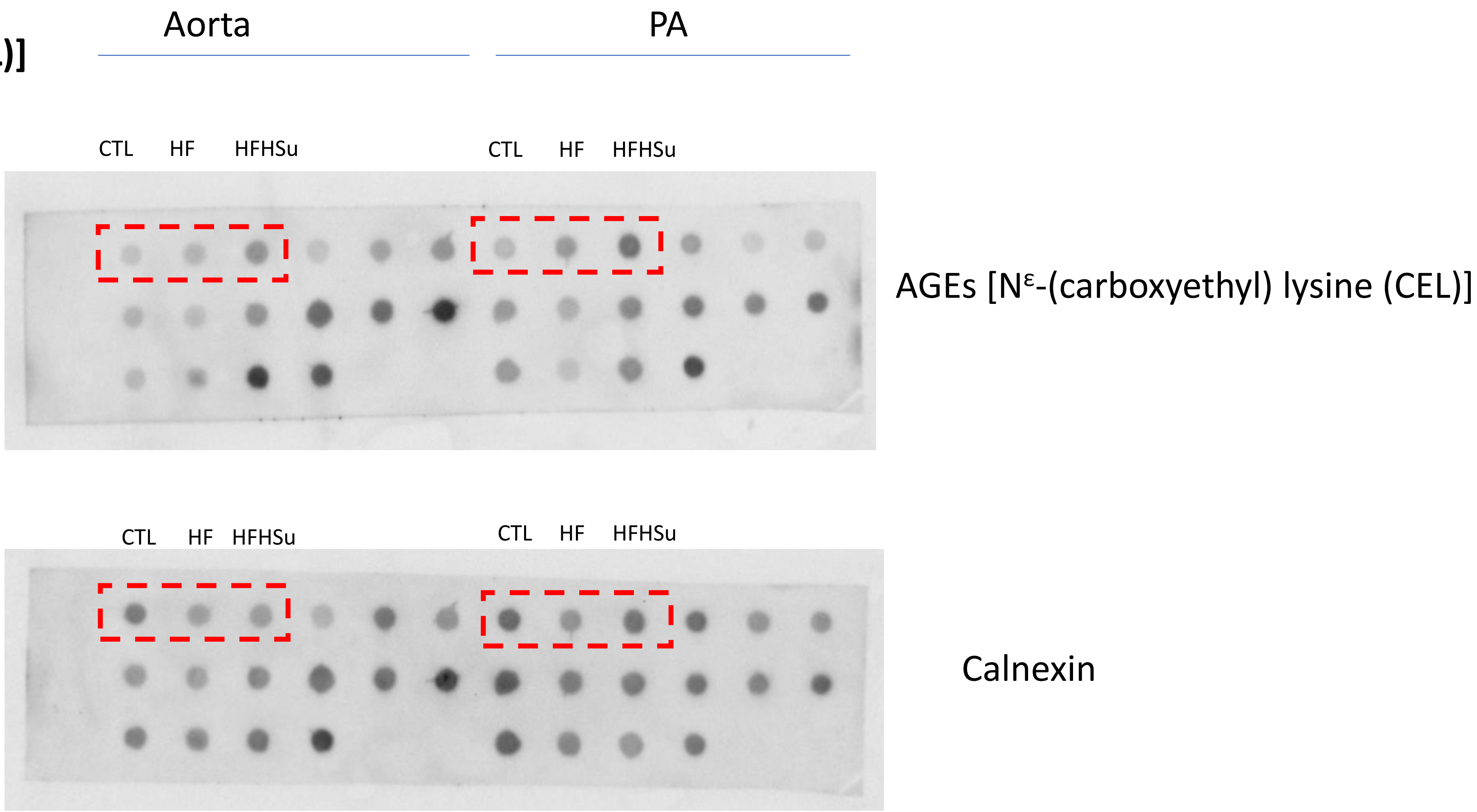

Figure 5 b

AGEs receptor

Aorta

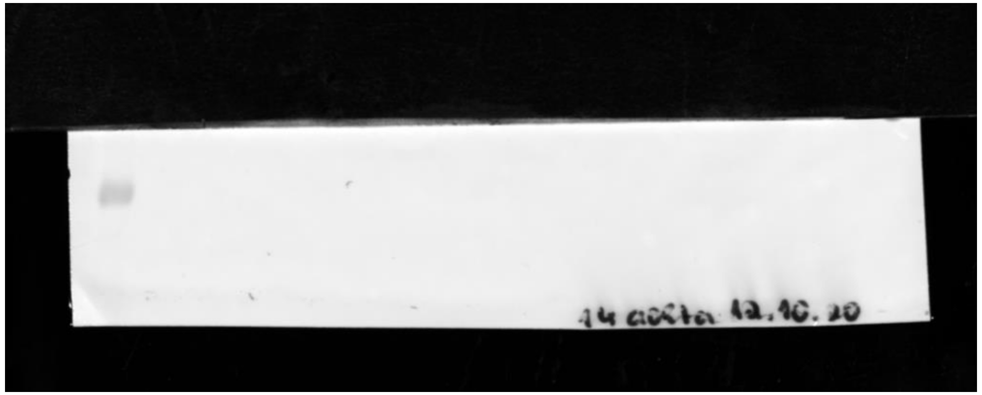

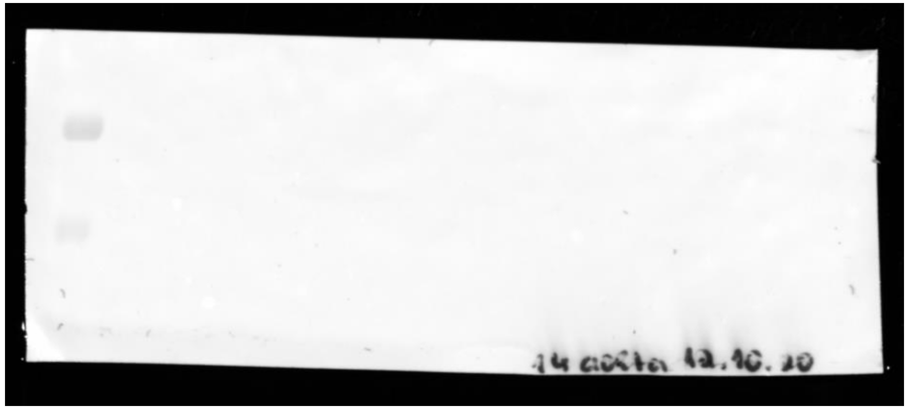

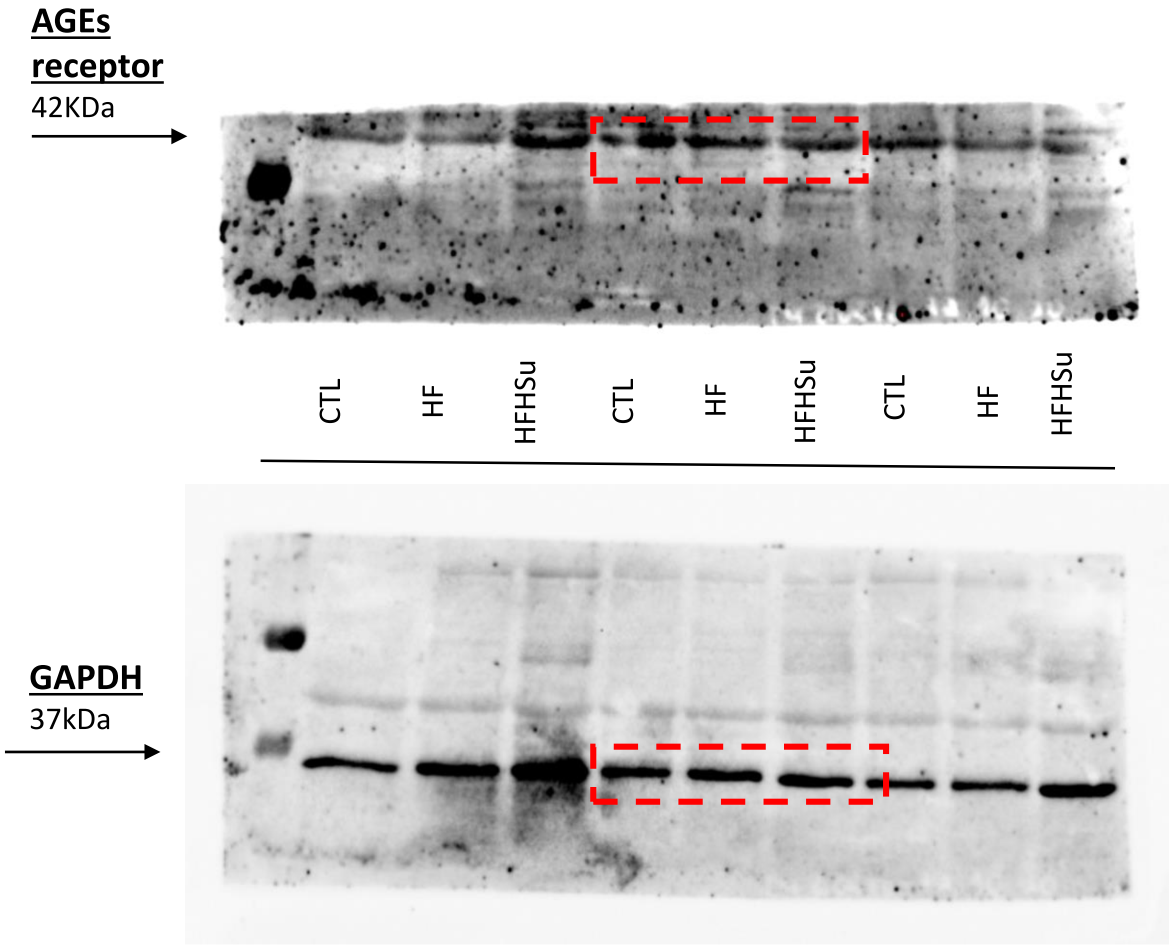

Figure 5 b

AGEs receptor

Pulmonary Artery

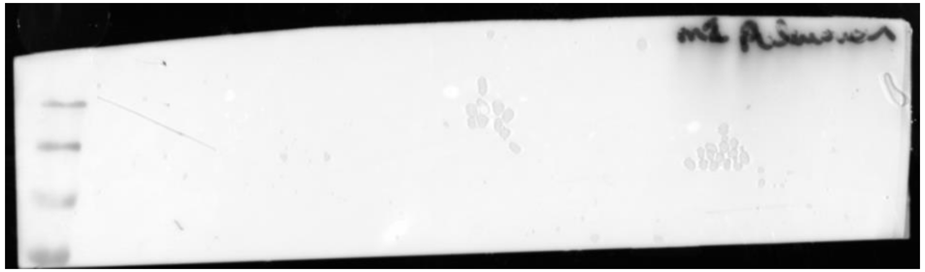

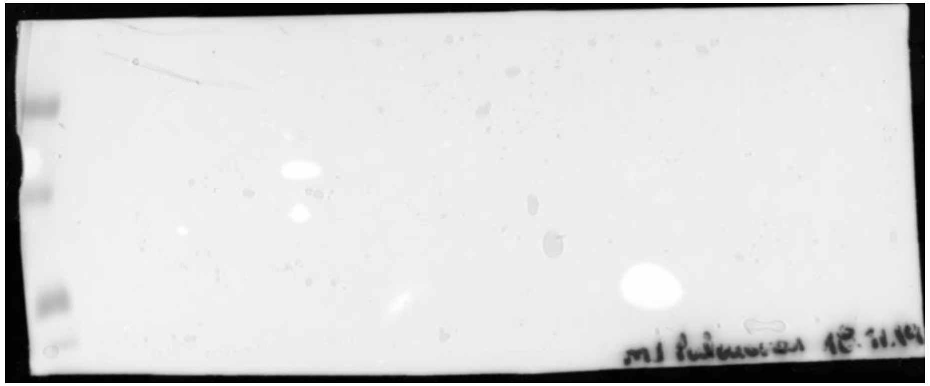

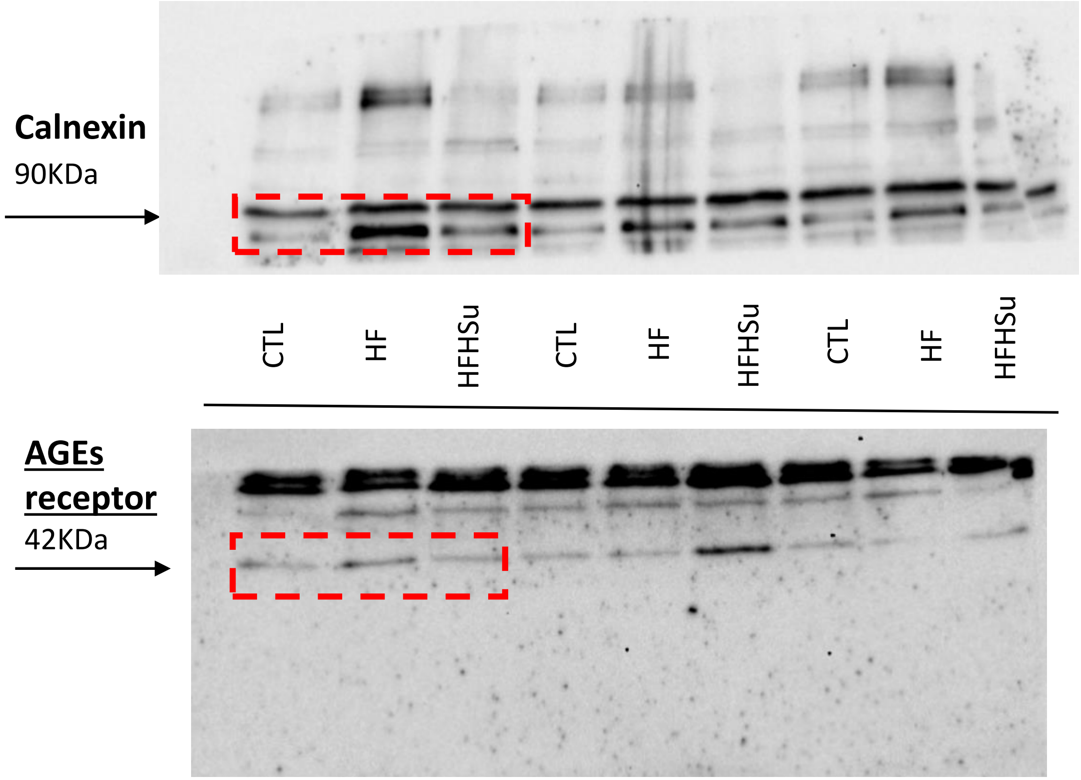

Figure 6 a

IL-1 receptor

Aorta

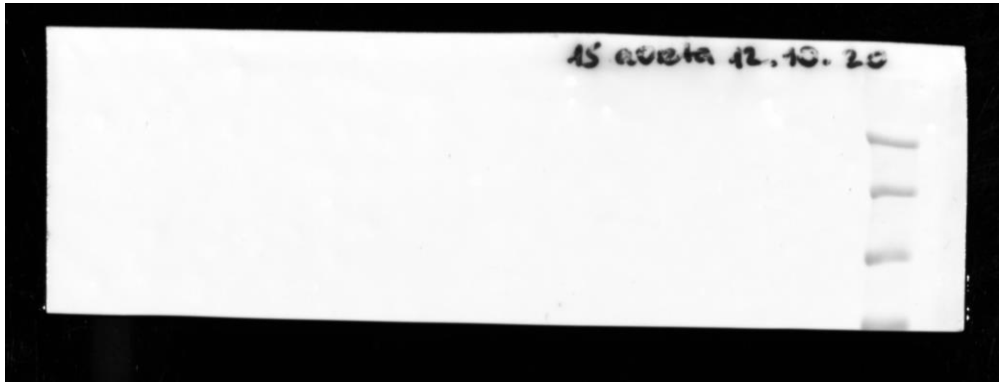

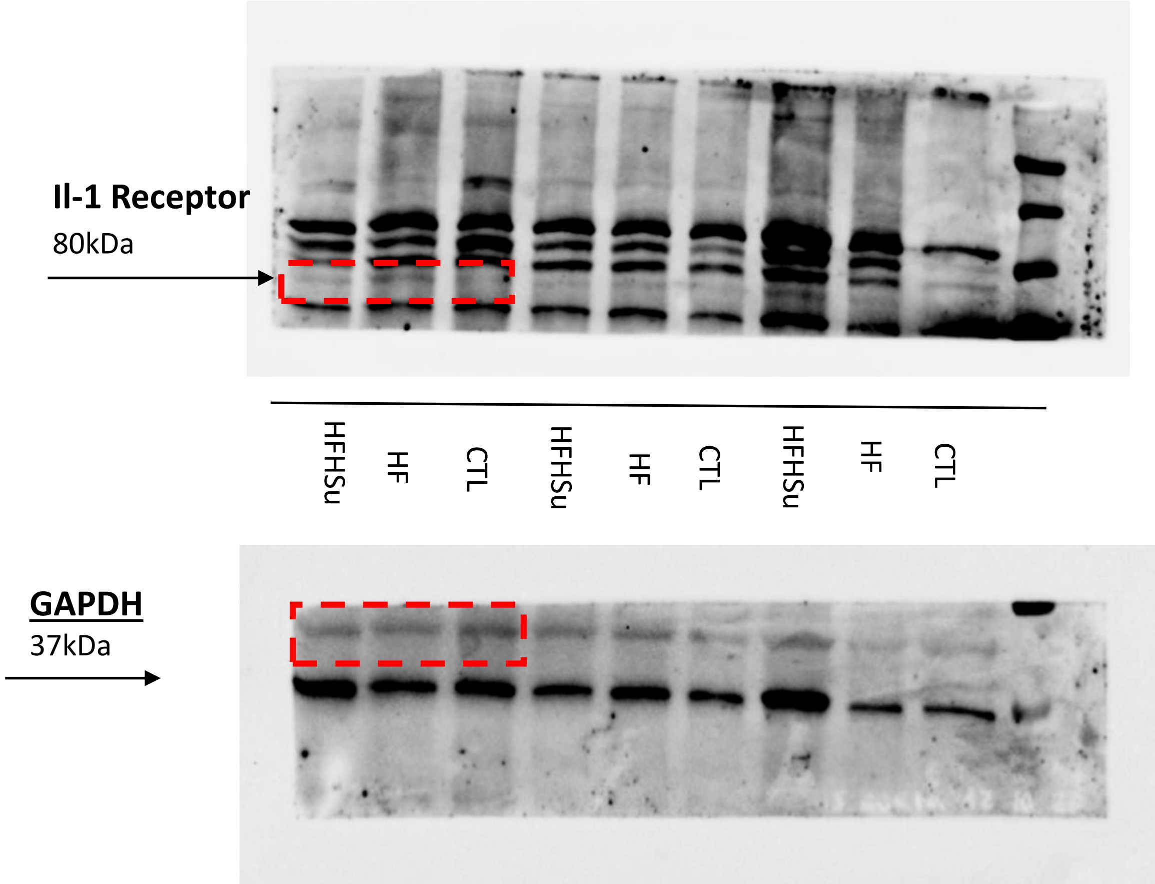

Representative bands cropped for the manuscript

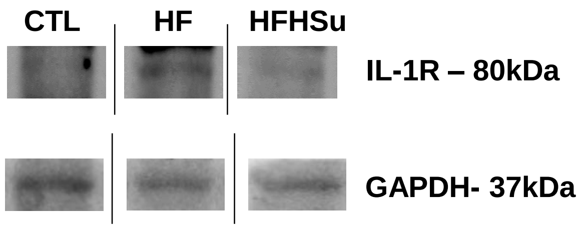

Figure 6 a

IL-1 receptor

Pulmonary artery

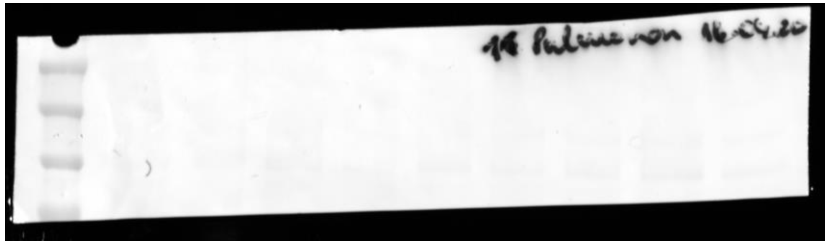

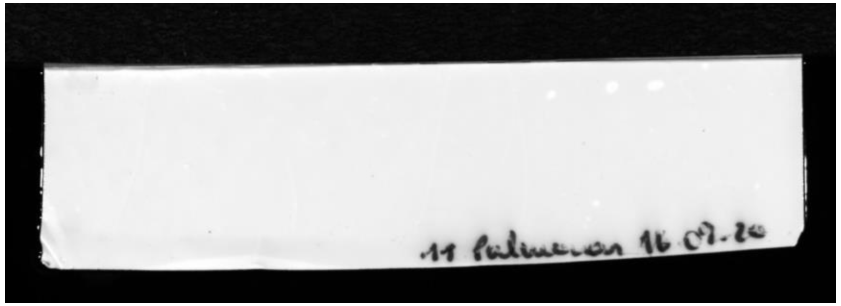

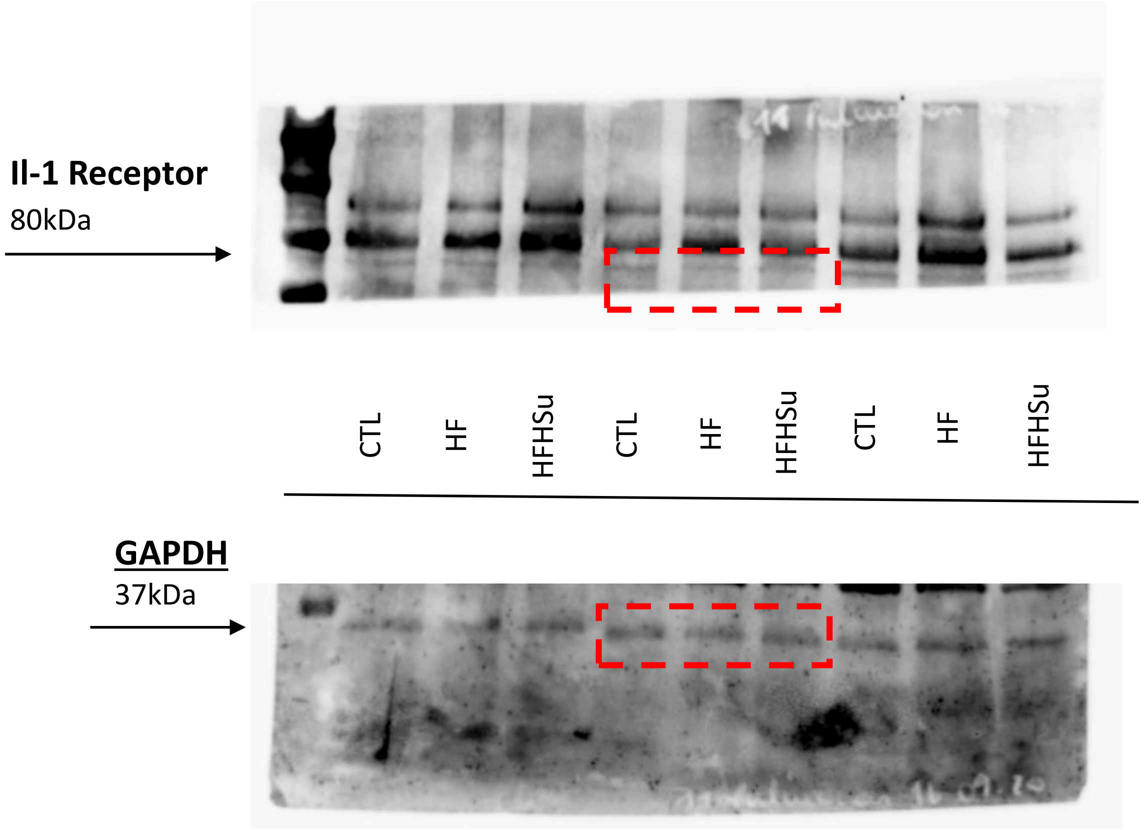

Figure 6 b

IL-6 receptor

Aorta

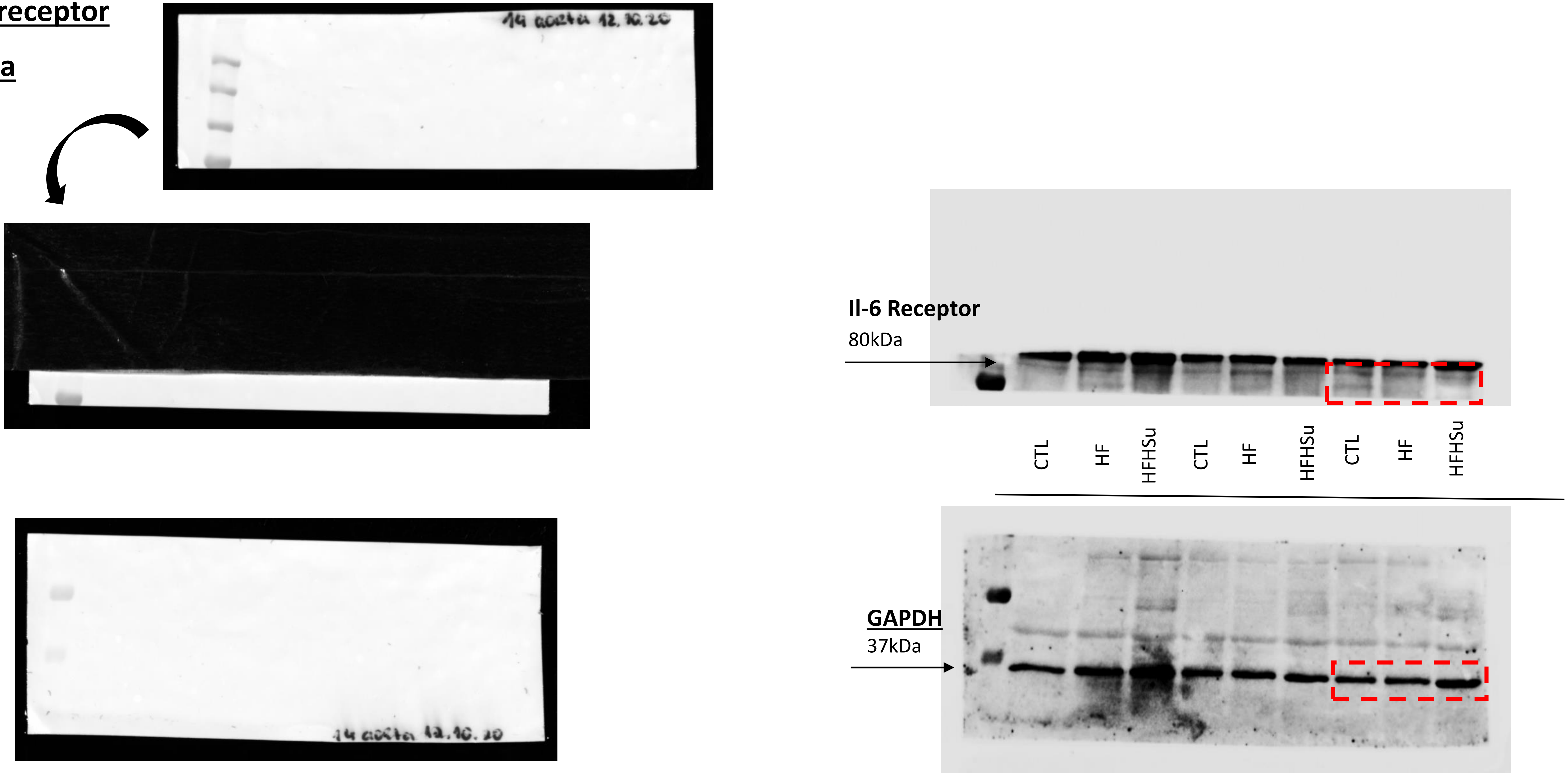

Figure 6 b

IL-6 receptor

Pulmonary artery

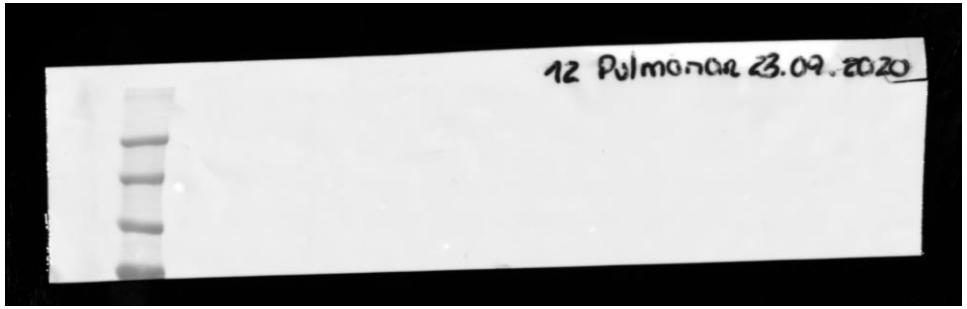

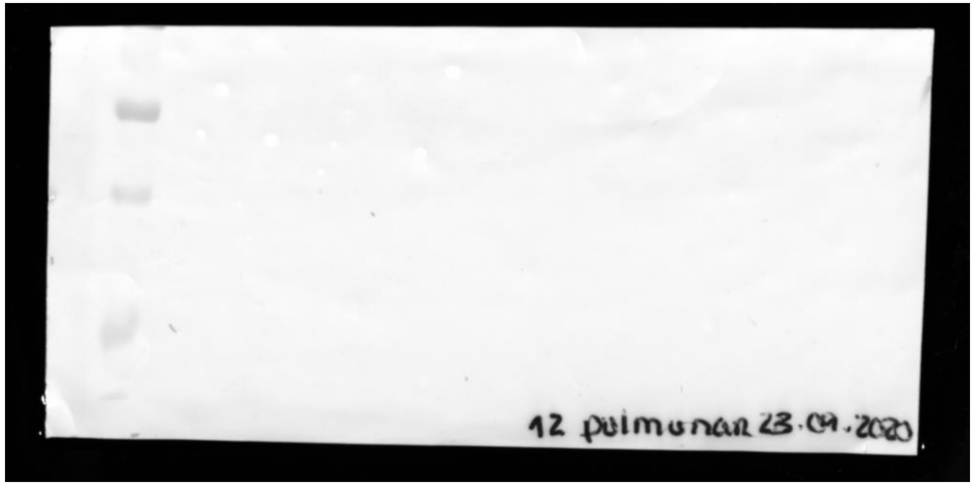

Il-6 Receptor
80kDa

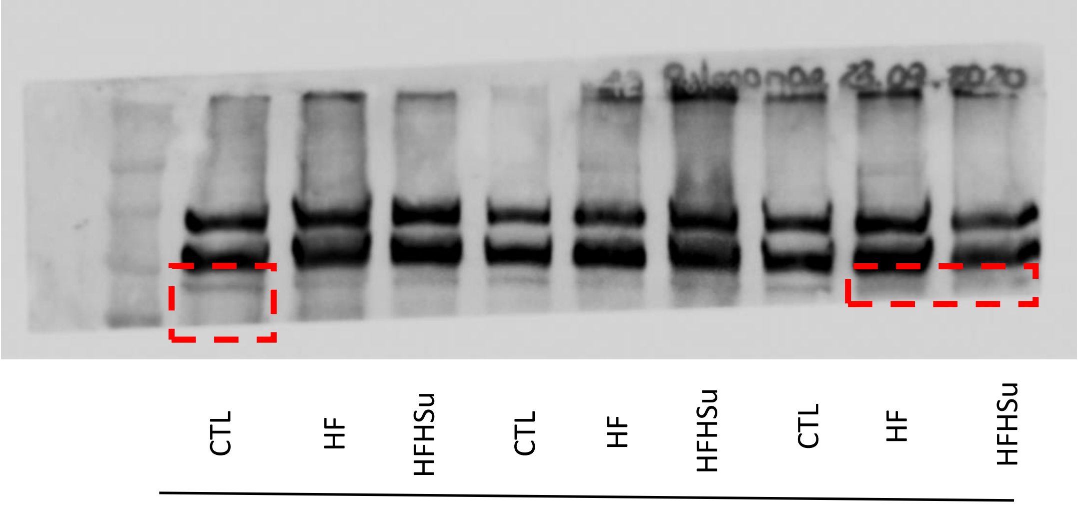

GAPDH
80kDa

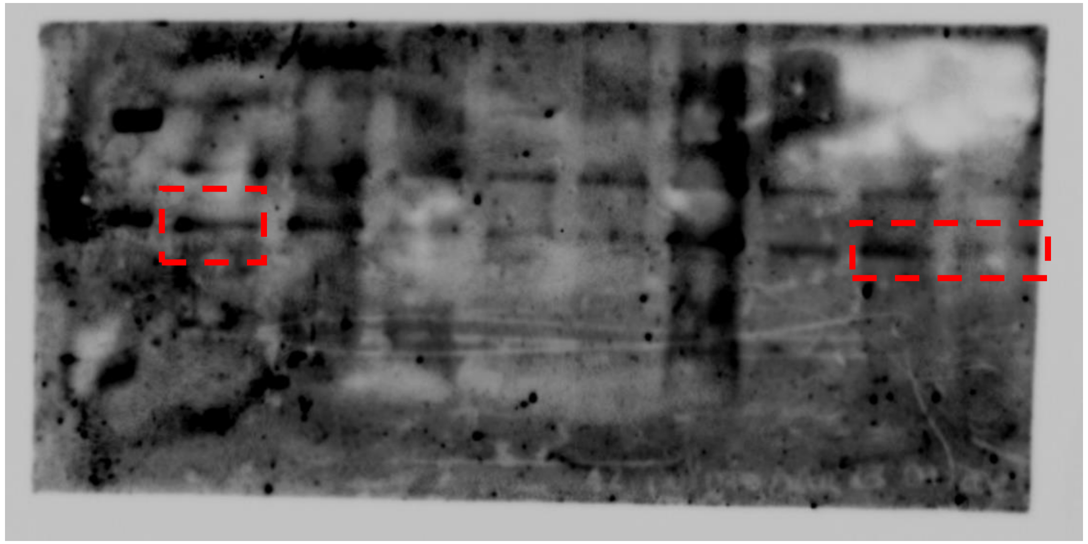

Representative bands cropped for the manuscript

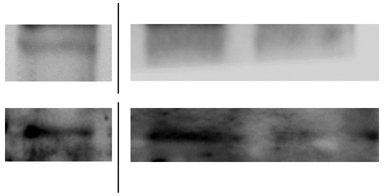

Il-6 Receptor
80kDa

GAPDH
37kDa

Figure 7

Catalase

Aorta

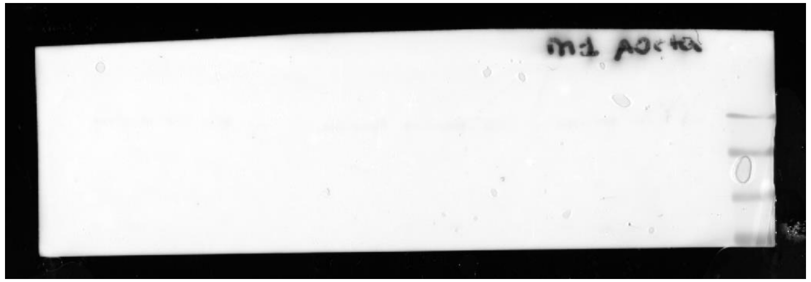

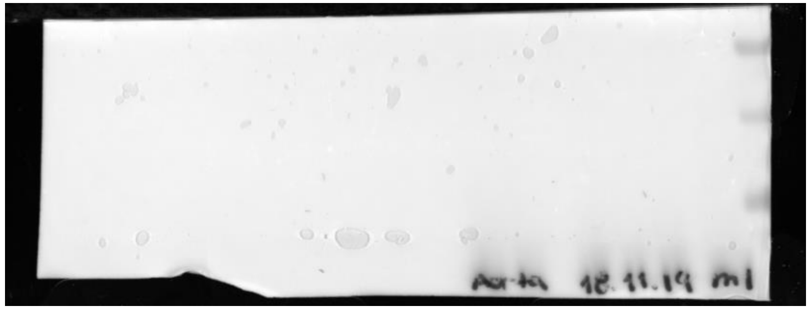

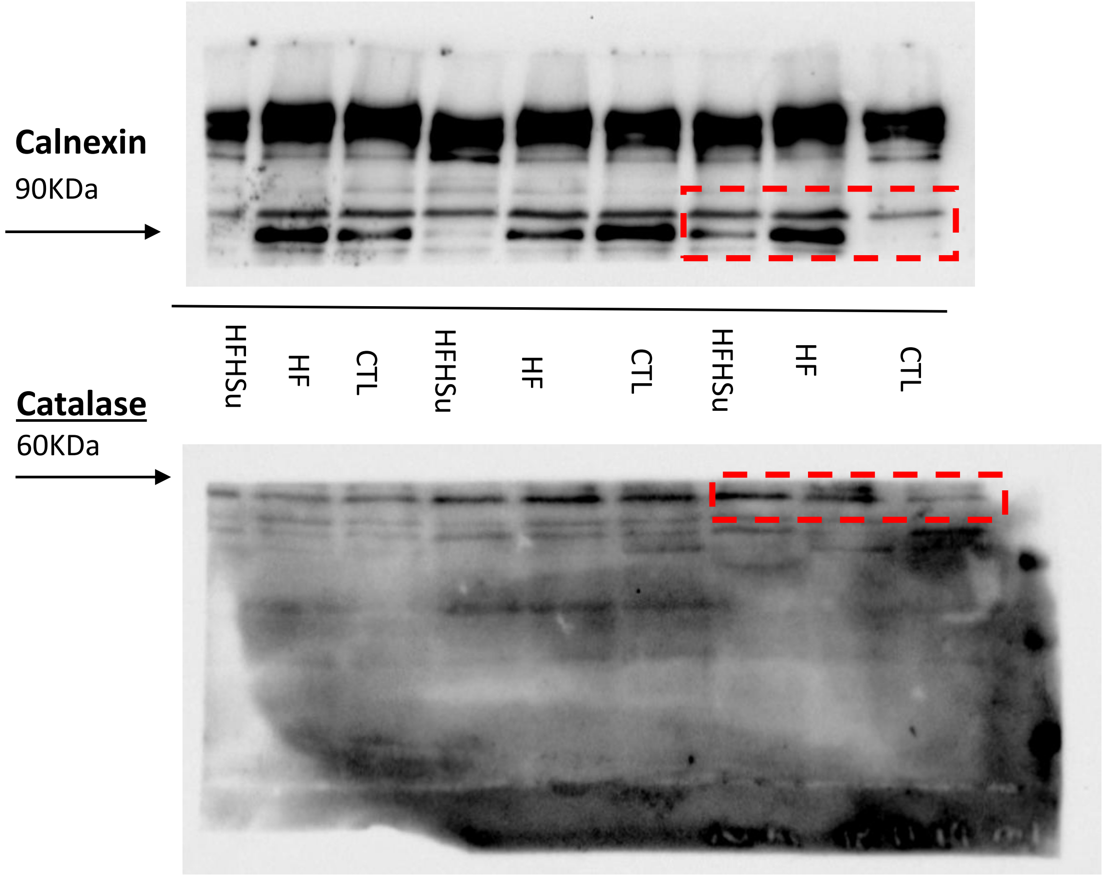

Representative bands cropped for the manuscript

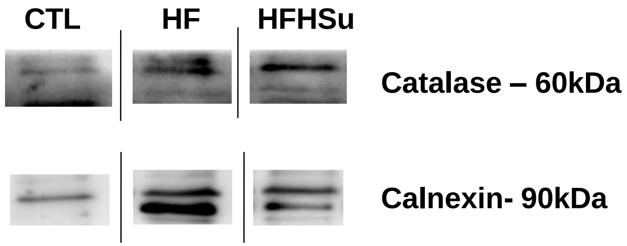

Figure 7

Catalase

Pulmonary Artery

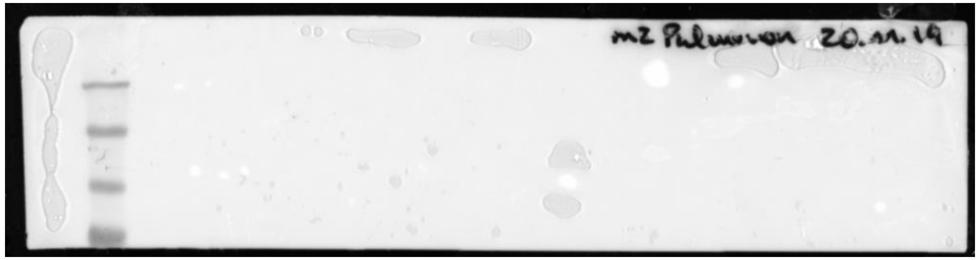

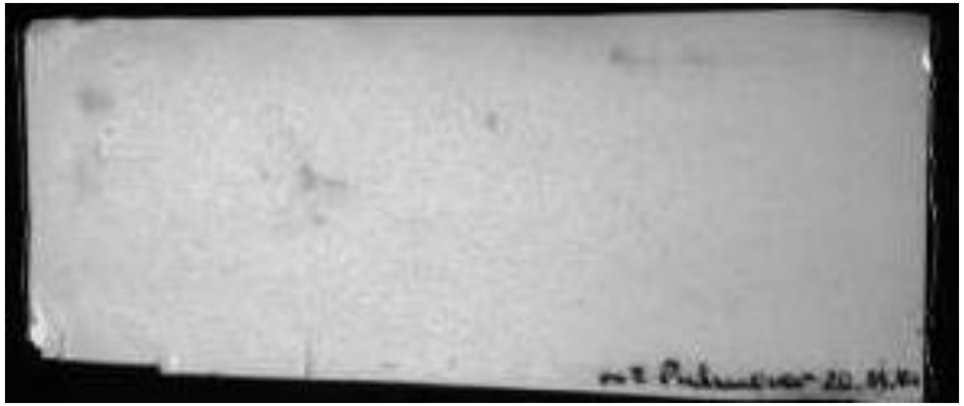

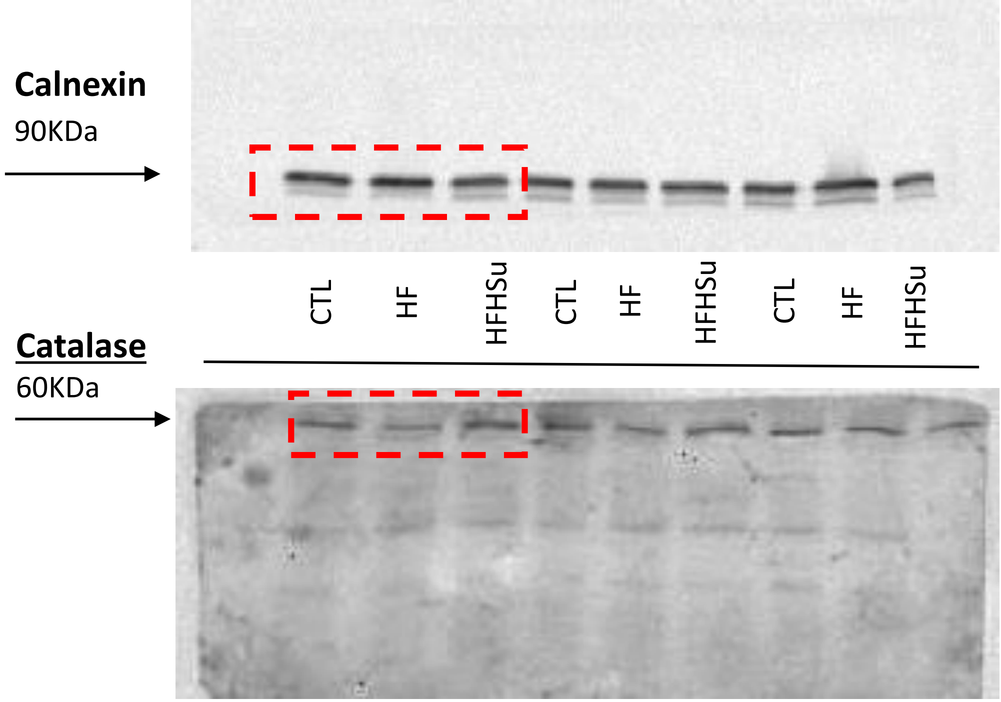

Supplement: Supplementary file 1 — Supplementary Information 1. [file 41598_2021_85606_MOESM1_ESM.pdf]
